# β-Caryophyllene Counteracts Chemoresistance Induced by Cigarette Smoke in Triple-Negative Breast Cancer MDA-MB-468 Cells

**DOI:** 10.3390/biomedicines10092257

**Published:** 2022-09-12

**Authors:** Antonella Di Sotto, Marco Gullì, Marco Minacori, Romina Mancinelli, Stefania Garzoli, Ester Percaccio, Alessio Incocciati, Donatella Romaniello, Gabriela Mazzanti, Margherita Eufemi, Silvia Di Giacomo

**Affiliations:** 1 Department of Physiology and Pharmacology “V. Erspamer”, Sapienza University of Rome, Piazzale Aldo Moro 5, 00185 Rome, Italy; 2 Department of Biochemical Science “A. Rossi Fanelli”, Sapienza University of Rome, Piazzale Aldo Moro 5, 00185 Rome, Italy; 3 Department of Anatomical, Histological, Forensic and Orthopedic Sciences, Sapienza University of Rome, Piazzale Aldo Moro 5, 00185 Rome, Italy; 4 Department of Chemistry and Technology of Drugs, Sapienza University of Rome, Piazzale Aldo Moro 5, 00185 Rome, Italy; 5 Department of Experimental, Diagnostic and Specialty Medicine, University of Bologna, 40138 Bologna, Italy

**Keywords:** cigarette smoke, caryophyllene sesquiterpenes, breast cancer, chemoresistance, STAT3, autophagy, γH2AX, cell migration, IL-8, environmental pollution

## Abstract

Exposure to cigarette smoke (CS) has been associated with an increased risk of fatal breast cancers and recurrence, along with chemoresistance and chemotherapy impairment. This strengthens the interest in chemopreventive agents to be exploited both in healthy and oncological subjects to prevent or repair CS damage. In the present study, we evaluated the chemopreventive properties of the natural sesquiterpene β-caryophyllene towards the damage induced by cigarette smoke condensate (CSC) in triple negative breast cancer MDA-MB-468 cells. Particularly, we assessed the ability of the sesquiterpene to interfere with the mechanisms exploited by CSC to promote cell survival and chemoresistance, including genomic instability, cell cycle progress, autophagy/apoptosis, cell migration and related pathways. β-Caryophyllene was found to be able to increase the CSC-induced death of MDA-MB-468 cells, likely triggering oxidative stress, cell cycle arrest and apoptosis; moreover, it hindered cell recovery, autophagy activation and cell migration; at last, a marked inhibition of the signal transducer and activator of transcription 3 (STAT3) activation was highlighted: this could represent a key mechanism of the chemoprevention by β-caryophyllene. Although further studies are required to confirm the in vivo efficacy of β-caryophyllene, the present results suggest a novel strategy to reduce the harmful effect of smoke in cancer patients and to improve the survival expectations in breast cancer women.

## 1. Introduction

Increasing epidemiological evidence has highlighted that the exposure to environmental pollution can increase the risk of various diseases, including cancer [1,2,3,4,5,6]; additionally, low-level pollutants have been shown to trigger cancer initiation and progression, and to promote tissue invasion, metastasis and chemoresistance [7,8,9]. Among the environmental toxicants, cigarette smoke (CS), both active and passive, is known to act as a promoting factor for the incidence of breast cancer, its aggressiveness, and recurrence [10,11,12,13,14,15]. Moreover, the risk of breast cancer was found to be enhanced in susceptible subjects exposed to environmental smoke and to E-cigarettes [16,17,18,19].

Although little is still known about the role of tobacco carcinogens in breast cancer progression and metastasis, nicotine seems to play a pivotal role, since it is able to promote cell proliferation and angiogenesis by modulating different cascades, which in turn control the expression of proapoptotic and antiapoptotic factors [3,20]. Moreover, it induces an immune-mediated inflammatory response through the regulation of the genes involved in cancer cell proliferation and migration [21]. Nicotine also seems to contribute to the chemoresistance induced by CS, likely inducing oxidative stress and the overexpression of the ATP-binding cassette (ABC) proteins [22,23,24,25]. Along with nicotine, polycyclic aromatic hydrocarbons have been found able to reduce the effectiveness of anticancer drugs, by promoting their enzymatic degradation [25]. This strengthens the interest in developing possible protective strategies to limit the CS injury due to both active smoking and involuntary exposure. Among possible interventions, chemopreventive agents attracted great attention due to their ability to both prevent genotoxicity in healthy people and in highly susceptible subjects, and also to interfere with the proliferation and progression of pre-existing cancer, thus being able to support chemotherapy and to avoid relapses [26]. However, effective chemopreventive strategies against cigarette smoke are not currently available, and those against breast cancer are limited owing to their possible side effects [26,27,28,29].

**Figure 1:**
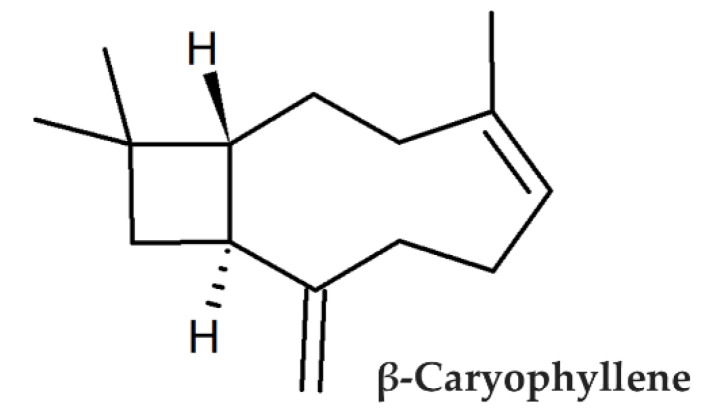
Chemical structure of β-caryophyllene (ChemSketch, Version 2018.1.1 free software, ACD/Labs, Toronto, ON, Canada).

Recent evidence highlighted the interesting chemopreventive and chemo-sensitizing properties of the natural caryophyllene sesquiterpenes β-caryophyllene (Figure 1) and its metabolite β-caryophyllene oxide; particularly, they prevented the damage of different environmental pollutants, including cigarette smoke, nitroarenes and alkylating agents, and sensitized different cancer cells to chemotherapy both in vitro and in vivo [26,30,31,32,33]. Among chemopreventive mechanisms, caryophyllene sesquiterpenes were able to interfere with DNA-damage, to modulate cell cycle progress and the cell redox state and inhibited the activation of the signal transducer and activator of transcription 3 (STAT3) pathway, thus in turn regulating the apoptotic cell death [26,32]. β-Caryophyllene also exhibited antiproliferative properties in triple negative breast cancer cells and modulated the expression of CB2 receptors [26,30,31].

In line with this evidence and in order to discover potential strategies to limit the harmful effect of smoke, in the present study we evaluated the ability of β-caryophyllene to counteract the chemoresistance induced by cigarette smoke condensate (CSC) in MDA-MB-468 triple-negative breast cancer cells. This cancer cell model was chosen due to the high risk of chemoresistance found in patients with triple-negative breast cancer and the associated aggressiveness and poor prognosis [34]. Different cell processes accounting for triple-negative breast cancer cell proliferation and progression, and probably responsible for chemoresistance, including autophagic/apoptotic cell death, oxidative stress, genomic instability, cell cycle progression and cell migration, were evaluated. In particular, cytoprotective autophagy is recognized as being a possible resistance mechanism in triple-negative breast cancer and its inhibition has been approached as an alternative adjuvant strategy to enhance chemotherapeutic efficacy [34]. Moreover, the genetic instability and deregulation of cell cycle checkpoints are known to favor the transformation processes, allowing the proliferation of altered cells under conditions inadequate for normal cells [35].

To perform the study, the key parameters known to trigger pro-survival autophagy in triple-negative breast cancer cells [36], including light chain (LC) 3II and beclin-1, and autophagosome formation were determined. Furthermore, the extent of apoptosis, whose interconnection with autophagy is widely documented, was studied. The intracellular levels of reactive oxygen species (ROS), reduced glutathione (GSH) and phosphorylated H2AX histone were assessed to monitor the cell redox state and genomic instability, respectively; cell cycle phase progression was also assessed. At last, the modulation of cell migration, and related factors, including BIRC-5 (baculoviral inhibitor of apoptosis repeat-containing 5 also known as survivin), MMP2 (matrix metalloproteinase-2), TPX2 (targeting protein for Xklp2) and interleukin (IL)-8 and STAT3, which is also a key control pathway for autophagy and apoptosis [37], were studied.

## 2. Materials and Methods

### 2.1. Chemicals

All of the chemicals, if not otherwise specified, were purchased from Merck Life Science S.r.l. (Milan, Italy); particularly, the β-caryophyllene was >98.0% purity. The sample of cigarette smoke condensate (CSC; batch n. R140701; 40 mg/mL of smoke particulates in DMSO), produced by smoking the University of Kentucky’s 3R4F reference cigarettes on a FTC Smoke Machine, was provided by Murty Pharmaceuticals Inc. (Lexington, KY, USA). Dulbecco’s Modified Eagle’s medium (DMEM), buffer, fetal bovine serum, and cofactors were from Aurogene S.r.l. (Rome, Italy). All of the solutions were prepared in the better solvent, sterilized by filtration and stored for a just conservation time at recommended temperature. The sesquiterpene β-caryophyllene was dissolved in ethanol (EtOH 100% *v*/*v*) while the CSC sample was dissolved in dimethyl sulfoxide (DMSO 100% *v*/*v*). The solvents were used at a maximum 1% *v*/*v* nontoxic concentration in the medium.

### 2.2. Chemical Analysis of Cigarette Smoke Condensate (CSC)

#### 2.2.1. Solid-Phase Micro Extraction (SPME)

To investigate the volatile chemical composition of cigarette smoke condensate (CSC), the solid-phase micro extraction (SPME) sampling technique was used. The sample was placed into a 7 mL glass vial with polytetrafluoroethylene (PTFE)-coated silicone septum. The samples were equilibrated for 30 min at 40 °C prior to analysis. For the sampling, a SPME device from Supelco (Bellefonte, PA, USA) with 1 cm fiber coated with 50/30 μm divinylbenzene/carboxen/polydimethylsiloxane (DVB/CAR/PDMS) was used. After an initial conditioning phase of the fiber, at 270 °C for 20 min, it was exposed to the equilibrated samples’ headspace for 120 min at 80 °C to capture the volatiles’ compounds. Later, the SPME fiber was inserted in a gas chromatography (GC) injector maintained at 250 °C for the desorption phase.

#### 2.2.2. Gas Chromatography and Mass Spectrometry (GC-MS) Analysis of CSC

GC-MS analysis was carried out on a Perkin Elmer Clarus 500 GC-MS and gas chromatography-free induction decay (GC-FID) (Waltham, MA, USA) equipped with a Varian VF-5ms (Factor Four of Superchrom) capillary column (60 m × 0.25 mm, film thickness 0.25 µm), according to previous methods [38] with some modifications. Injector temperature was 250 °C, detector temperature 300 °C, while the column temperature was held at 50 °C and programmed to 170 °C at a rate of 6 °C/min then increased to 270 °C at a rate of 8 °C/min and then kept isothermally at 270 °C for 10 min; helium was used as the carrier gas with 1 mL/min flow; ionization energy was 70 eV and mass ranged from 35 to 450 amu. The components were identified by comparison of their mass spectra to those reported in the Wiley and NIST/NBS (National Institute of Standards and Technology/National Bureau of Standards) libraries. Furthermore, the Linear Retention Indices (LRIs) were calculated and compared with the available retention data reported in the literature. The peak areas of the FID signal were used to calculate the relative percentages of the components without the use of an internal standard and any factor correction. All of the analyses were carried out in triplicate.

### 2.3. Cell Line

Triple-negative breast cancer MDA-MB468 cells were obtained from the IST National Institute for Cancer Research, Advanced Biotechnology Center/Interlab Cell Line Collection (CBA/ICLC) (Genoa, Italy) and grown at 37 °C in 5% CO_2_ in DMEM, supplemented with fetal bovine serum (10% *v*/*v*), L-glutamine (2 mM), streptomycin (100 µg/mL), and penicillin (100 U/mL). All of the experiments were performed when the cells reached the logarithmic growth phase.

### 2.4. Trypan Blue Exclusion Test of Cell Viability

Confluent cells (5 × 10^4^ cells) were seeded in 24-well plates, then treated with β-caryophyllene and CSC for the required time exposure. At the end of the treatments, the cells were harvested and the cell suspension stained by trypan blue and examined visually under light microscopy using a hemocytometer to determine whether the cells included or excluded the stain. Nonviable cell and viable cells were identified on the basis of a blue and clear cytoplasm, respectively. After cell counting, the total number of viable cells was determined according to previous methods [39].

### 2.5. Cytotoxicity Assay

Confluent cells were seeded into 96-well microplates (2 × 10^4^ cells/well), allowed to grow for 24 h, then subjected to a 24 h treatment with progressive dilutions of the test substances (CSC, 5, 10, 50, 75, 100 and 150 µg/mL; β-caryophyllene, 1, 5, 10, 15, 20, 25 and 50 µg/mL). After incubation, the cytotoxicity was measured by the 3-[4,5-dimethylthiazol-2-yl]-2,5-diphenyl tetrazolium bromide (MTT) assay, using an Epoch Microplate Spectrophotometer (BioTek, AHSI, Milan, Italy) [32]. Cytotoxicity was determined as at least a 30% lowering of cell viability with respect to the control [40].

### 2.6. Treatment Schedules Applied to Evaluate the Chemopreventive Properties of β-Caryophyllene towards CSC

**Figure 2:**
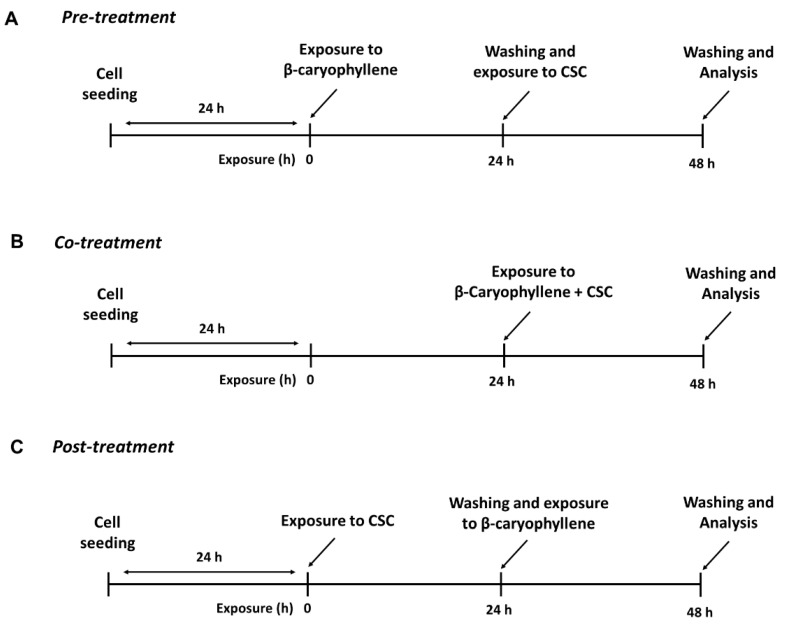
Scheduled treatment protocols applied to evaluate the chemopreventive properties of β-caryophyllene towards cigarette smoke condensate (CSC) in triple-negative breast cancer MDA-MB-468 cells: pre-treatment (**A**), co-treatment (**B**) and post-treatment (**C**).

To perform the study, the cells (2 × 10^4^ cells/well) were exposed to nontoxic concentrations of β-caryophyllene and CSC under three different treatment protocols, i.e., pre-, co- and post-treatments, as follows. In the pre-treatment protocol, the cells were incubated overnight with β-caryophyllene, then washed and treated with CSC for a further 24 h; in the co-treatment protocol, the cells were exposed to a combination of β-caryophyllene and CSC for 24 h; last, in the post-treatment, the cells were treated overnight with CSC, then with β-caryophyllene for a further 24 h after CSC removal (Figure 2). At the end of the treatments, the cells and the relative supernatants underwent analysis.

**Figure 3:**
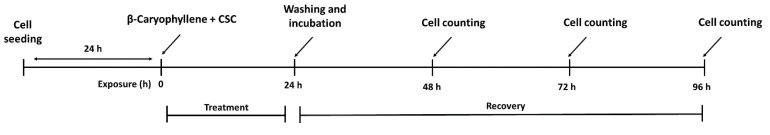
Scheme of the treatment protocol applied to evaluate the recovery abilities of triple-negative breast cancer MDA-MB-468 cells after a 24 h exposure to β-caryophyllene and cigarette smoke condensate (CSC) alone and in co-treatment.

A further exposure protocol was applied to evaluate the recovery abilities of the cells after treatments (Figure 3). To this end, the cells were treated with β-caryophyllene and CSC for 24 h, then washed and allowed to recover for further 72 h. Cell proliferation was monitored during recovery and the proliferation index was determined. At last, suitable time exposures were applied to better highlight specific parameters, such as apoptosis and autophagy, as explained in the relative paragraphs.

### 2.7. Determination of Lactate Dehydrogenase (LDH) Release

The assay was performed using a Roche LDH Cytotoxicity Detection Kit (Sigma-Aldrich, Milan, Italy), according to the manufacturer’s instructions. To perform the assay, the confluent cells (2 × 10^4^ cells/well) were exposed to nontoxic concentrations of β-caryophyllene and CSC under the scheduled treatment protocols, then incubated as previously reported. After incubation, the cell supernatants were harvested and centrifuged to remove debris, then mixed with the assay reagent and incubated for 30 min away from light; after incubation, the absorbance was read spectrophotometrically at 490 nm using an Epoch Microplate Spectrophotometer (BioTek, AHSI, Milan, Italy). The LDH levels were normalized per number of viable cells.

### 2.8. Intracellular Reactive Oxygen Species (ROS) Determination

ROS levels were measured by the 2,7-dichlorofluorescein diacetate assay (DCFH-DA), according to a previous method with minor changes [41]. Fluorescence was measured at excitation and emission wavelengths of 485 nm and 535 nm, using a Cytation 1 Cell Imaging Multimode Reader (BioTek, AHSI, Milan, Italy). The results were normalized to viable cells and expressed as a percentage of the negative control. A fluorescence imaging analysis of the treated, methanol-fixed and dichlorofluorescein (DCF)-stained cells, using a Cytation 1 Cell Imaging Multimode Reader (BioTek, AHSI, Milan, Italy) was also performed.

### 2.9. Determination of Intracellular Glutathione Levels

A standardized chromatographic HPLC-UV method was applied to determine the intracellular levels of reduced (GSH) and oxidized (GSSG) glutathione, as previously described [32]. The ratio between the GSH and GSSG (expressed as μM per mg of protein) was compared among the treatments.

### 2.10. Detection of Phosphorylated Histone H2AX (γH2AX)

The levels of phosphorylated H2AX at serine 129 (γH2AX), as a measure of the DNA damage induced by treatments, were detected by fluorescence imaging analysis using a Cytation 1 Cell Imaging Multimode Reader (BioTek, AHSI, Milan, Italy). To this end, 5 × 10^4^ cells were seeded in 24-well plates, then treated with β-caryophyllene and CSC for the required time exposure. At the end of the treatments, the cells were fixed in pure methanol and stained by an Alexa Fluor*^®^* 647 anti-gamma H2A.X (phospho S139) antibody (Abcam; Cod. ab195189, Aurogene S.r.l., Rome, Italy) and Hoechst 33,258 (1 µg/mL) dye. The fluorescence intensity was measured by the Gen5™ Microplate Reader and Imager Software, (Version 3.11, BioTek, AHSI, Milan, Italy) and expressed with respect to the cell numbers.

### 2.11. Cell Cycle Analysis

The analysis of the cell cycle distribution was evaluated by fluorescence imaging analysis of DNA content using a Cytation 1 Cell Imaging Multimode Reader (BioTek, AHSI, Milan, Italy), according to a previous method with minor changes [42]. To this end, the cells were treated as reported for the γH2AX analysis, then fixed in pure methanol and stained by Hoechst 33,258 (1 µg/mL) dye, which selectively binds DNA into nucleus, leading to a total fluorescence intensity proportionate to the DNA content of cell. Fluorescence images were captured and deconvoluted by Gen5™ Microplate Reader and Imager Software (Version 3.11, BioTek, AHSI, Milan, Italy); moreover, the fluorescence intensity of the Hoechst 33,258-stained nuclei and the relative area were determined. At least 500 cells per images were evaluated and the histograms relating the total fluorescence to the % count were generated using Gen5™ Microplate Reader and Imager Software (Version 3.11, BioTek, AHSI, Milan, Italy), according to the protocol described by the producer [43]. The histogram plots were subsequently used to identify G1 and G2 cell cycle phases, based on the prior biological knowledge that the DNA content of G2 cells is doubled with respect to the G1 cells, while the S-phase cells were in the intervening region between the G1 and G2 cells.

### 2.12. Apoptosis Detection

The extent of apoptosis was revealed through Annexin-V staining and measured by both flow cytometry and fluorescence microscopy, according to the method reported by Di Sotto et al. [32]. To perform the assay, the cells were seeded in 24-well plates (1 × 10^5^ cells/well), subjected to treatments with β-caryophyllene and CSC, then collected for the flow cytometric analysis; in each experiment, a vehicle control, corresponding to a basal apoptosis rate, was also included. The apoptotic and viable cells were revealed by a flow cytometric Annexin-V-Cy3 detection kit (Sigma-Aldrich, Milan, Italy) and the mean fluorescence of 50,000 cells was determined by a BD AccuriTM C6 Software (Version 1.0.264.21, BD Biosciences, Milan, Italy). A multiparameter fluorescence analysis and the gating of forward and side scatter (FSC-SSC) were performed by the BD AccuriTM C6 Software (Version 1.0.264.21, BD Biosciences, Milan, Italy). Furthermore, the treated cells, fixed in pure methanol and stained by Annexin-V- fluorescein isothiocyanate (FITC) and Hoechst 33,258 (1 µg/mL) dyes, were also subjected to a fluorescence imaging analysis using a Cytation 1 Cell Imaging Multimode Reader (BioTek, AHSI, Milan, Italy). Trypan blue exclusion test allowed for the distinguishing of apoptosis from necrosis, and to show membrane integrity after Annexin-V binding. Fluorescence intensity was measured by the Gen5™ Microplate Reader and Imager Software (Version 3.11, BioTek, AHSI, Milan, Italy), and expressed with respect to the cell numbers.

### 2.13. Autophagy Detection

The analysis was executed using an Autophagy Assay Kit (Sigma-Aldrich MAK138, Milan, Italy), according to the manufacturer’s instructions. Briefly, 2 × 10^5^ cells were treated with β-caryophyllene and CSC, then collected for the flow cytometric analysis; in each experiment, a vehicle control, corresponding to a basal autophagy rate, was also included. After the treatments, the cell medium was replaced by an autophagosome detection reagent solution (1×; 100 µL) for 1 h; thereafter, the cells were washed and the fluorescence intensity measured at an excitation wavelength of 360 nm and an emission of 420 nm by a Appliskan^®^ multimode microplate reader (Thermo Scientific, Waltham, MA, USA). Autophagy was also detected in the treated, methanol-fixed and stained cells by fluorescence imaging analysis, using a Cytation 1 Cell Imaging Multimode Reader (BioTek, AHSI, Milan, Italy).

### 2.14. Immunofluorescence Analysis of Autophagy Factors LC3 and Beclin-1

Immunofluorescence analysis was performed according to a previous published method [32]. Briefly, the cells, seeded on coverslip in a six-well plate and subjected to treatments, were fixed in methanol, washed in Phosphate Buffered Saline + Tween 20 (PBS-T) and incubated in 4% bovine serum albumin (BSA) and PBS-T, then further incubated with the LC3 and Beclin-1 primary antibodies for 1 h at room temperature (RT). After washing in PBS-T, the cells were placed in the specific Alexa Fluor 488 secondary antibody (Invitrogen, Thermo Fisher Scientific, Waltham, Massachusetts, USA) for 45 min in a dark room at RT and rinsed with PBS-T, then a coverslip was put onto slide with a drop of 4′,6-Diamidino-2-phenylindole dihydrochloride (DAPI). The slides were examined to analyze LC3 and Beclin-1 expression by Leica Microsystems DM 4500 B Light and Fluorescence Microscopy (Leica Microsystems, Weltzlar, Germany) equipped with a JenoptikProg-Res-C10 Plus Videocam (Jenoptik Optical Systems GmbH, Jena, Germany). A semiquantitative analysis (four fields for each treatment) for the intensity of the fluorescence was completed according to the following grading system [44]: negative, <5%; +/−, 6–10%; +, 11–30%; ++, 31–60%; +++, >61%.

### 2.15. Cell Migration through Wound Healing Assay

The confluent monolayer cells were grown in 12-well microplates (5 × 10^5^ cells/well) for 24 h, then subjected to a mechanical injury by a sterile p-10 pipette tip, to obtain a vertical wound for each well, as previously reported [41]. Thereafter, the cells were treated with nontoxic concentrations of β-caryophyllene and CSC under the scheduled treatment protocol. High-contrast bright-field images of the growing cells were captured at zero time and after 24 h and 48 h incubation, using a Cytation 1 Cell Imaging Multimode Reader (BioTek, AHSI, Milan, Italy). The area of the wound was quantified by a Gen5™ Microplate Reader and Imager Software (Version 3.11, BioTek, AHSI, Milan, Italy).

### 2.16. RT-qPCR Analysis of Gene Expression of Cell Migration Factors

RT-qPCR (Real-Time Quantitative Polymerase Chain Reaction) analysis was carried out according to a previous method [45], using suitable oligonucleotides for BIRC-5, MMP2, TPX2 and IL-8 (Bio-Rad, Hercules, CA, USA), a MJ Mini Opticon Detection System (Bio-Rad, Hercules, CA, USA) with SYBR green fluorophore and Brilliant SYBR Green QPCR Master Mix (Thermo Fisher Scientific, Monza, Italy). Gene expression analysis was performed by CFX Manager^TM^ Real Time PCR Detection System Software (Version 3.1, Bio-Rad, Hercules, CA, USA), using the glyceraldehyde 3-phosphate dehydrogenase (GAPDH) (Bio-Rad, Hercules, CA, USA) gene for normalization. Details of the oligonucleotides are displayed in Table 1.

### 2.17. Western Blotting Analysis of STAT3

The confluent cells (5 × 10^5^ cells/well) were treated as described above, then harvested and lysed using a suitable buffer for Western blotting analysis, according to previous methods [32]. Signals were acquired by a Molecular Imager^®^ ChemiDoc™ MP System (Bio-Rad, Hercules, CA, USA), and the intensity of protein bands quantified by the ImageJ software (Version 1.52n, National Institutes of Health, Bethesda, MD, USA). The levels of phospho(Tyr705)-STAT3 were normalized against total STAT3 ones; β-actin (total extracts) was also included as the normalization protein.

### 2.18. Statistical Analysis

All of the data are expressed as mean ± standard error (SE) of at least two biological replicates in which at least two technical replicates per each concentration were performed. The statistical analysis was carried out by GraphPad Prism™ software (Version 6.00, GraphPad Software, Inc., San Diego, CA, USA). The one-way analysis of variance (one-way ANOVA), followed by Dunnett’s Multiple Comparison Post-Test, was used to analyze the difference between treatments. The concentration–response curves were constructed using the “Hill equation”, according to previous methods [32]. A *p* value < 0.05 was considered as significant.

## 3. Results

### 3.1. Volatile Chemical Profile of Cigarette Smoke Condensate (CSC)

SPME-GC/MS analysis allowed for the revelation of the presence of eight volatile compounds in the CSC sample, as shown in Table 2; among them, neophytadiene (56.4%) was the most abundant, followed by nicotine (26.7%) and 7-methyl-6-tridecene (6.9%); other minor compounds, including α-myrcene, triacetin, (Z)-2,6,10-trimethyl-1,5,9-undecatriene, 6,11-dimethyl-2,6,10-dodecatrien-1-ol and urs-12-en-28-ol, were found with relative percentages from 0.8% to 3.1%.

### 3.2. Effects of β-Caryophyllene and Cigarette Smoke Condensate (CSC) on Viability and Recovery of Triple Negative Breast Cancer MDA-MB-468 Cells

**Figure 4:**
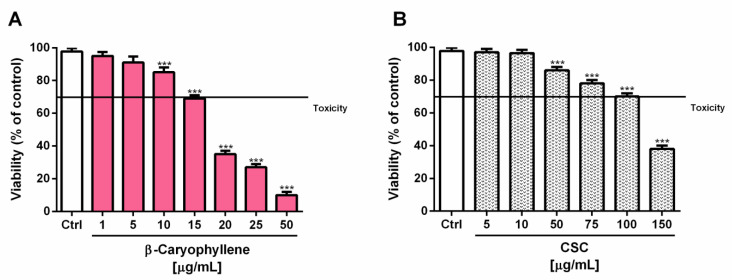
Cytotoxic effects of β-caryophyllene (**A**) and cigarette smoke condensate; CSC (**B**) in triple-negative breast cancer MDA-MB-468 cells after 24 h exposure. Data are displayed as mean ± SE of at least three independent experiments with at least three technical replicates (*n* = 9). *** *p* < 0.001, significant lowering of cell viability with respect to the control (ANOVA + Dunnett’s multiple comparison post-hoc test).

Preliminary cytotoxicity assays were performed to evaluate the effects of β-caryophyllene and CSC in triple-negative breast cancer MDA-MB-468 cells after 24 h exposure and to select the nontoxic concentrations to be assessed for the chemopreventive studies. According to our previous study [30], β-caryophyllene was cytotoxic starting from the concentration of 20 µg/mL (about 60% inhibition of cell viability), with early toxicity signs at 15 µg/mL (about 30% inhibition of cell viability) (Figure 4A).

Similarly, CSC produced concentration-dependent cytotoxic effects, with early signs (about 30% inhibition of cell viability) at the concentration of 100 µg/mL (Figure 4B). In terms of potency, the IC_50_ values showed that β-caryophyllene was about seven-fold more toxic than CSC (Table 3); as expected, the positive control doxorubicin was almost four-fold more cytotoxic than β-caryophyllene under the same experimental conditions.

**Figure 5:**
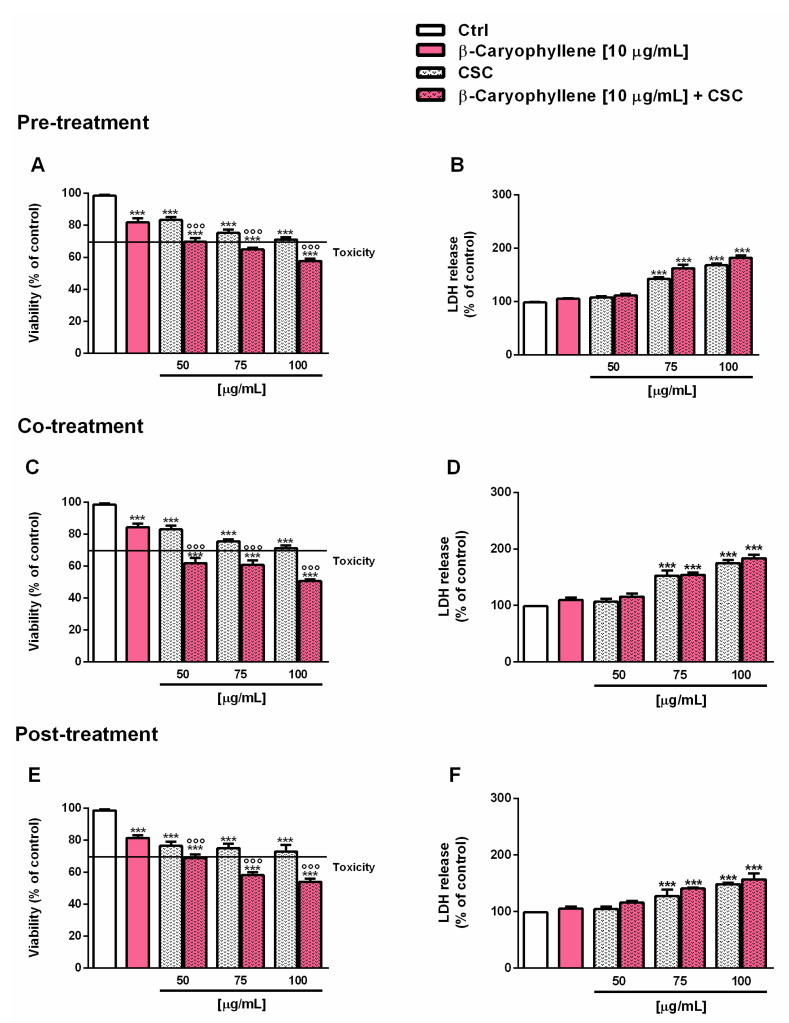
Cytotoxic effects of β-caryophyllene and cigarette smoke condensate (CSC) in triple-negative breast cancer MDA-MB-468 cells under pre-, co- and post-treatment protocols, measured as cell viability (A, C, E) and LDH release (B, D, F). Data are displayed as mean ± SE of at least three independent experiments with at least three technical replicates (*n* = 9). *** *p* < 0.001, significant lowering of cell viability with respect to the control (ANOVA + Dunnett’s multiple comparison post-hoc test). °°° *p* < 0.001, significant difference the respect to CSC (Student’s *t*-test).

Based on the results of the cytotoxicity assay, the concentration of 10 µg/mL, which induced a lower than 20% cytotoxicity, was selected for studying the chemopreventive properties of β-caryophyllene towards CSC under different scheduled protocols of pre-, co- and post-treatment. These conditions were evaluated in line with our previous studies in which the substance was shown to modulate the injury of cigarette smoke and cigarette butt extract, acting as both a desmutagenic and a bioantimutagenic agent [33,46]. In particular, the modulating effects of the sesquiterpene were assessed towards three nontoxic or low-toxic concentrations of CSC (i.e., 50, 75 and 100 µg/mL) and the cytotoxicity was determined in terms of both the cell viability and leakage of the cytoplasmic lactate dehydrogenase (LDH) enzyme. This last assay provided information about the ability of the treatment to damage the cell membrane, causing the release of the intracellular contents, including the detected enzyme: this event is usually correlated with necrosis occurrence which in turn evokes an inflammatory response [47]. Under our experimental conditions, we found a similar behavior of the tested samples in the pre-, co- and post treatments. In particular, starting from the concentration of 75 µg/mL, the cytotoxicity of CSC was associated with a progressive increase in LDH leakage, which was not significantly affected by the treatment with β-caryophyllene (Figure 5). At the concentration of 50 µg/mL, CSC produced a cytotoxic effect lower than or equal to 20% with respect to the control, which was further increased by the treatment with the sesquiterpene up to a maximum of 20% in the co-treatment, without affecting LDH release (Figure 5).

On the basis of these findings, the co-treatment of 10 µg/mL β-caryophyllene and 50 µg/mL CSC was chosen for further evaluating the recovery abilities of MDA-MB-468 cells and the modulation of chemoresistance mechanisms. The cell number was registered at the end of the treatment; in addition, the recovery abilities of triple-negative breast cancer MDA-MB-468 cells after 24 h exposure to the co-treatment were evaluated every 24 h for a total of 72 h and expressed as the recovery index (i.e., the ratio between the cell numbers at each time point of recovery and that after treatment).

**Figure 6:**
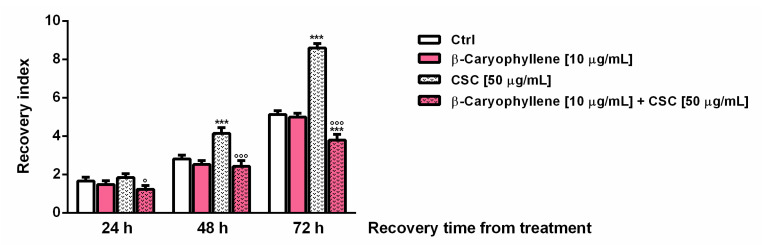
Effect of the co-treatment of β-caryophyllene and cigarette smoke condensate (CSC) in triple-negative breast cancer MDA-MB-468 cells after 24 h exposure and subsequent 72 h cell recovery. Recovery abilities were expressed as a recovery index, which represents the ratio between the cell number at each time point of recovery and that achieved after 24 h treatment. Cell number was determined by trypan blue exclusion assay at each time point. Data are displayed as mean ± SE of at least two experiments with at least three technical replicates (*n* = 6). *** *p* < 0.001, significant lowering of cell viability the respect to the control (ANOVA + Dunnett’s multiple comparison post-hoc test). ° *p* < 0.05 and °°° *p* < 0.001, significant difference the respect to CSC (Student’s *t*-test).

The results highlighted that, after a significant lowering of cell viability induced by treatments, the cell growth progressively increased, especially in the control and in CSC-treated cells (Figure S1). The recovery index of the cells subjected to CSC treatment was found markedly increased by about two- and three-fold after 48 and 72 h recovery, respectively, whereas β-caryophyllene inhibited the CSC effect, bringing the index back to the control levels (Figure 6); moreover, after 72 h recovery, a further 30% lowering with respect to the control was also highlighted. This evidence suggested that, along with an initial injury by CSC, the tumor-promoting factors can be activated by the toxicant, leading to increased cell recovery abilities and probably to chemoresistance; conversely, β-caryophyllene seemed to counteract the activation of these processes acting as a chemo-preventing agent.

In order to clarify the phenomenon and to better understand the processes responsible for CSC-induced chemoresistance and for the protective effects of β-caryophyllene, different cell parameters, including the redox state, genomic instability, autophagic and apoptotic rates, and cell migration were studied under the selected experimental conditions, as explained in the following sections.

### 3.3. Effects of β-Caryophyllene and Cigarette Smoke Condensate (CSC) on Intracellular Oxidative Stress

**Figure 7:**
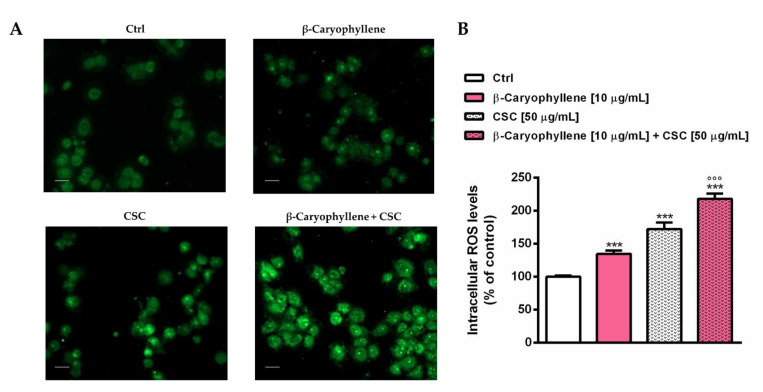
Intracellular ROS levels induced by β-caryophyllene and cigarette smoke condensate (CSC) in MDA-MB-468 cancer cells after 24 h exposure. (**A**) Representative images of cells stained by DCF. Original magnification 10×. (**B**) Bar graph of DCF-fluorescence. Data are displayed as mean ± SE of at least three independent experiments with at least three technical replicates (*n* = 9). *** *p* < 0.001, significant lowering of cell viability with respect to the control (ANOVA + Dunnett’s multiple comparison post-hoc test). °°° *p* < 0.001, significant difference with respect to CSC (Student’s *t*-test). Scale bars = 20 μm.

The redox state of triple-negative breast cancer MDA-MB-468 cells subjected to a 24 h co-treatment with β-caryophyllene and CSC was determined in terms of levels of reactive oxygen species (ROS) and reduced glutathione (GSH). The results highlighted the ability of both β-caryophyllene and CSC to upregulate the intracellular oxidative stress, as shown by the increased ROS levels induced by both the tested samples and their combination (Figure 7). At imaging analysis, the oxidative stress was highlighted by the green DCF-fluorescence into the cells, as displayed in Figure 7A. Quantification of the DCF-fluorescence showed that the ROS levels were enhanced by about 30% by β-caryophyllene, and by at least 70% by CSC, while more than doubled by their co-treatment (Figure 7B). The increased oxidative stress can be responsible for the activation of cell-death signaling, thus explaining the higher cytotoxicity highlighted by the co-treatment with β-caryophyllene and CSC with respect to the samples alone in all of the assessed protocols of exposure.

**Figure 8:**
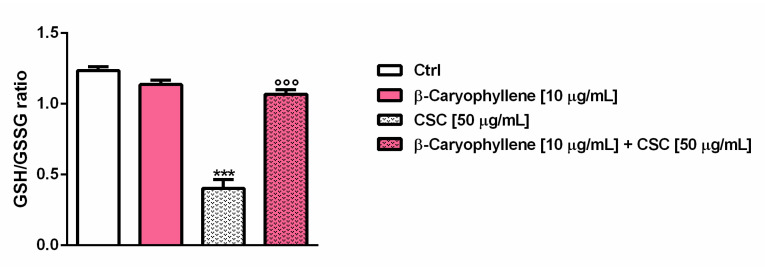
Bar graphs representing the levels of GSH (reduced glutathione) and GSSG (oxidized glutathione) induced by β-caryophyllene and cigarette smoke condensate (CSC) in MDA-MB-468 cancer cells after 24 h exposure as revealed by HPLC analysis. Data are displayed as mean ± SE of at least three independent experiments with at least two technical replicates (*n* = 6). *** *p* < 0.001, significant lowering of cell viability with respect to the control (ANOVA + Dunnett’s multiple comparison post-hoc test). °°° *p* < 0.001, significant difference with respect to CSC (Student’s *t*-test).

According to the marked oxidative stress, CSC induced a three-fold reduction of the ratio between the GSH levels and GSSG levels, despite a slight lowering (about 1.09 fold) found with β-caryophyllene; conversely, the co-treatment of β-caryophyllene and CSC almost restored the GSH/GSSG ratio, inducing about a 2.7-fold increase (Figure 8). These results suggest that the treatment with β-caryophyllene only slightly affected the glutathione levels, despite a marked depletion (about a four-fold lowering with respect to the control) induced by CSC. However, considering that the GSSG amount was similar to that of the control, GSH depletion by CSC could be due to other mechanism than ROS species neutralization, such as conjugation of the toxicants contained in the smoke mixture.

### 3.4. Effects of β-Caryophyllene and Cigarette Smoke Condensate (CSC) on H2AX Histone Phosphorylation and Cell Cycle Modulation

**Figure 9:**
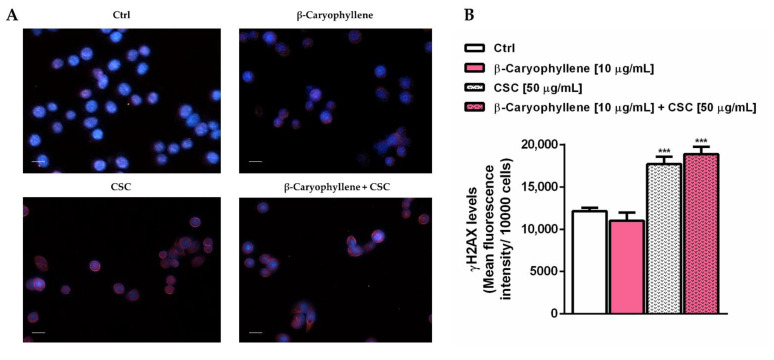
Levels of γH2AX induced by cigarette smoke condensate (CSC) and β-caryophyllene in MDA-MB-468 cancer cells after 24 h exposure. (**A**) Representative images of cells stained by Alexa Fluor^®^ 647 anti-gamma H2A.X (phospho S139) antibody and Hoechst 33,258 dye. (**B**) Data are displayed as mean ± SE of at least three independent experiments with at least two technical replicates (*n* = 6). *** *p* < 0.001, significant lowering of cell viability with respect to the control (ANOVA + Dunnett’s multiple comparison post-hoc test). Scale bars = 20 μm.

Under our experimental conditions, we also evaluated the genomic stability of triple-negative breast cancer MDA-MB-468 cells, which represents a key feature of the different cancer cells, by measuring the levels of phosphorylated H2AX histone (*γ*H2AX). Moreover, considering that genomic instability and altered cell cycle are often responsible for deregulated cancer cell proliferation and invasion, we also assessed the modulation of cell cycle phases by β-caryophyllene and CSC, as a possible mechanism accounting for the chemopreventive properties of β-caryophyllene towards cigarette smoke. According to the enhanced oxidative stress, the levels of *γ*H2AX were found increased by CSC by at least 30%, without alterations due to the presence of β-caryophyllene (Figure 9); a similar trend was also found under pre-treatment and post-treatment exposures (Figure S2). At imaging analysis, the increase in the *γ*H2AX levels were highlighted by the red fluorescence around the cells (Figure 9A). The lacking genoprotective effects of β-caryophyllene towards CSC in MDA-MB-468 cells could be ascribed to the inefficacy of DNA repair systems in restoring DNA integrity, leading to damage accumulation and cell-death activation.

In line with this evidence, we also evaluated the role of β-caryophyllene in the control of cell cycle progression of the CSC-damaged MDA-MB-468 cells, which could promote the activation of cell death signaling in the presence of unsolved DNA damage.

**Figure 10:**
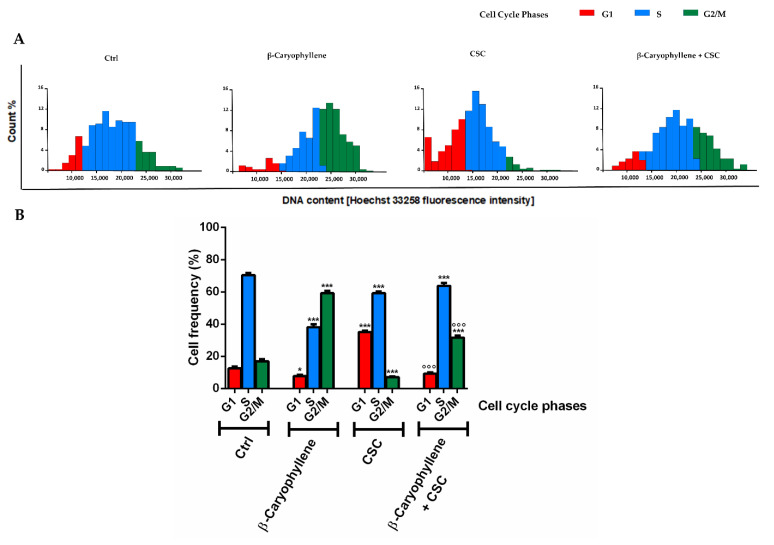
Modulation of cell cycle phases induced by β-caryophyllene and cigarette smoke condensate (CSC) in MDA-MB-468 cancer cells after 24 h exposure. (**A**) Histograms showing the percentages of cells stained by Hoechst 33,258 in different cell cycle phases under the scheduled treatments. (**B**) Data are displayed as mean ± SE of at least three independent experiments with at least three technical replicates (*n* = 9). * *p* < 0.05 and *** *p* < 0.001, significant lowering of cell viability with respect to the control (ANOVA + Dunnett’s multiple comparison post-hoc test). °°° *p* < 0.001, significant difference with respect to CSC (Student’s *t*-test).

As displayed by both the histograms and graph bars (Figure 10A,B), β-caryophyllene produced almost a 40% reduction in the S cell cycle phase along with a slight but significant lowering of the G1 phase with respect to the control; moreover, it enhanced the G2/M phase by about 67% with respect to the control (Figure 10A,B). CSC stimulated the cell accumulation in the G1 phase, with about a 63% increase with respect to the control, while the S and G2/M phases were almost 14% and 60% lower than the control. This suggests higher proliferative abilities of the CSC-stimulated cells and a reduced control in the G2/M phase which allow damaged cells to escape. Conversely, when CSC was assessed in the presence of β-caryophyllene, the G1 phase was markedly reduced (about a three-fold lowering) with respect to CSC, the G2/M phase almost quadrupled with a slight increase in the S phase. Moreover, comparing cell cycle distribution in the co-treatment of β-caryophyllene and CSC with respect to the control, the G1 phase results at least halved, while the G2/M phase almost doubled, with a slight lowering of the S phase. These findings suggest that β-caryophyllene induces a G2/M phase arrest in MDA-MB-468 cells which can lower their progression and also activate checkpoints, thus promoting cell death: this hypothesis agrees with the previous evidence in cholangiocarcinoma cells [32].

### 3.5. Effects of β-Caryophyllene and Cigarette Smoke Condensate (CSC) on Apoptosis and Autophagic Cell Death

**Figure 11:**
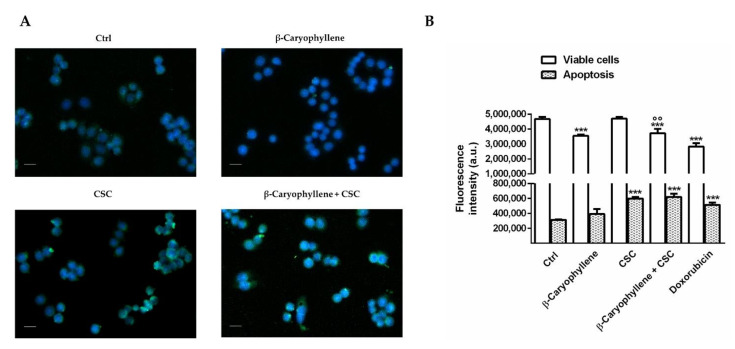
Apoptosis rate induced by β-caryophyllene, cigarette smoke condensate (CSC) and the positive control doxorubicin in MDA-MB-468 cancer cells after 24 h exposure. (**A**) Representative images of cells stained by Annexin V and Hoechst 33,258 dye. (**B**) Data are displayed as mean ± SE of at least three independent experiments with at least three technical replicates (*n* = 9). *** *p* < 0.001, significant lowering of cell viability with respect to the control (ANOVA + Dunnett’s multiple comparison post-hoc test). °° *p* < 0.01, significant difference with respect to CSC (Student’s *t*-test). Scale bars = 20 μm.

In order to clarify the cell death mechanisms involved in the increased cytotoxicity induced by the co-treatment of β-caryophyllene and CSC, the apoptosis rate was determined by both immunofluorescence and flow cytometric analysis (Figure 11). In particular, imaging was executed by staining cells with a Annexin-V-FITC probe and apoptosis was revealed as a green fluorescence around the cells. For the flow cytometric analysis, we exploited a previously published method [32], which allows for the detection of both apoptotic and viable cells, using Annexin-V-Cy3 and carboxyfluorescein diacetate (CFDA). The live and apoptotic cells were selected by their typical cell size and granularity, measured through the forward light scatter (FSC) and side scatter (SSC). Under our experimental condition, immunofluorescence analysis highlighted the presence of apoptosis (green fluorescence around the cells) in the cells treated with both CSC and CSC plus β-caryophyllene, without signs in the other treatments (Figure 11A). Flow cytometric analysis confirmed the imaging results and highlighted an increased apoptosis rate by about 50% with CSC and by about 40% with the co-treatment of CSC and β-caryophyllene; moreover, a slight 10% lowering of viable cells was induced by both β-caryophyllene and CSC, achieving 25% in combination (Figure 11B). As expected, the positive control doxorubicin lowered the viable cell rate by about 30% and increased apoptosis by at least 40% (Figure 11B). An early apoptosis activation was also found after 3 h exposure at imaging analysis; in these conditions, the effect of the co-treatment of β-caryophyllene and CSC appeared slightly higher than that induced by CSC (Figure S3), suggesting that apoptosis could be quickly induced, as a consequence of the marked oxidative and genotoxic damage and of the activation of cell cycle checkpoints.

**Figure 12:**
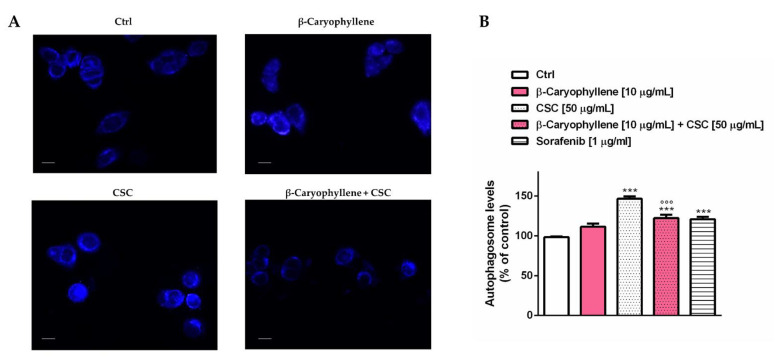
Autophagic cell death induced by β-caryophyllene, cigarette smoke condensate (CSC) and the positive control sorafenib in MDA-MB-468 cancer cells after 24 h exposure. (**A**) Representative images of cells stained by autophagosome detection reagent. (**B**) Bar graphs of fluorescence intensity as determined by quantitative analysis using autophagosome detection reagent. Data are displayed as mean ± SE of at least three independent experiments with at least three technical replicates (*n* = 9). *** *p* < 0.001, significant lowering of cell viability with respect to the control (ANOVA + Dunnett’s multiple comparison post-hoc test). °°° *p* < 0.001, significant difference with respect to CSC (Student’s *t*-test). Scale bars = 10 μm.

**Figure 13:**
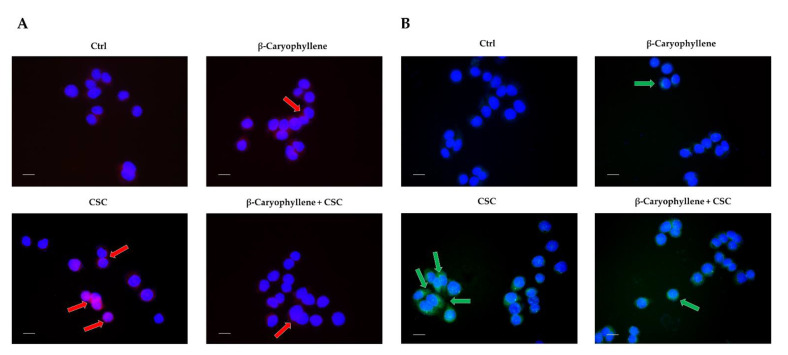
Immunofluorescence analysis of Beclin-1 and LC3 II expression induced by β-caryophyllene and cigarette smoke condensate (CSC) in MDA-MB-468 cancer cells after 24 h exposure. (**A**) Representative images of cells stained by a primary anti-Beclin 1 antibody, nucleic acid DAPI dye and secondary red fluorescent antibody. (**B**) Representative images of cells stained by a primary anti-LC3 II antibody, nucleic acid DAPI dye and secondary green fluorescent antibody. Data are displayed as mean ± SE of at least three independent experiments with at least three technical replicates (*n* = 9). Scale bars = 20 μm.

Under our experimental conditions, we also evaluated the rate of autophagic cell death, which is known to be involved in chemoresistance, especially for triple-negative breast cancer. To this end, the autophagosome formation and expression of autophagic factors were measured (Figure 12 and Figure 13). In particular, the autophagosome levels were significantly increased by CSC and the positive control sorafenib with respect to the control, while reduced by the presence of β-caryophyllene with respect to CSC; β-caryophyllene induced a slight but not significant increase in autophagosomes (Figure 12A). Quantification analysis showed that CSC induced a 50% increase in autophagy, which was reduced by at least 25% by β-caryophyllene. The effect of the co-treatment of β-caryophyllene and CSC was similar to that of the positive control sorafenib (Figure 12B).

According to these results, we also evaluated at immunofluorescence analysis the expression of the autophagic factors Beclin 1 (or BECN1) and LC3 II, which play a crucial role in autophagy regulation and autophagosome formation [36]. The presence of both factors in the cells was highlighted by a red and green fluorescence, respectively. Under our experimental conditions, we found that CSC markedly increased the expression of both Beclin 1 and LC3, which are two factors involved in autophagosome formation. Conversely, their levels were lowered by the co-treatment with β-caryophyllene (Figure 13A,B; Table 4). Altogether, these results suggest that inhibiting autophagy induced by CSC can represent a chemoprevention mechanism by which β-caryophyllene counteracts the chemoresistance induced by this toxicant to promote cell survival and progression. Moreover, it does not interfere with apoptosis but the process could be speeded up by β-caryophyllene, probably as a consequence of a cell cycle arrest and checkpoint activation.

### 3.6. Effects of β-Caryophyllene and CSC on Cell Migration and on Cell Migration Factors

**Figure 14:**
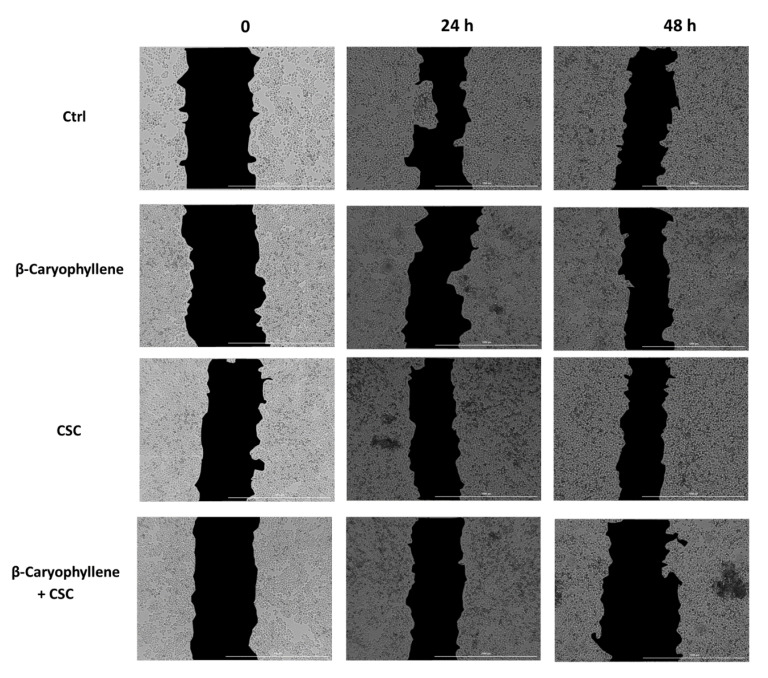
MDA-MB-468 cells migration rate at 0 h, 4 h and 72 h (*n* = 3) after co-treatment with β-caryophyllene and cigarette smoke condensate (CSC) in MDA-MB-468 cancer cells. A. Images of wound healing assay captured at each time point and processed by Gen5™ Microplate Reader and Imager Software (Version 3.11, BioTek, AHSI, Milan, Italy) (4× original magnification). The quantified wound areas are displayed in black. Scale bars = 1000 μm.

**Figure 15:**
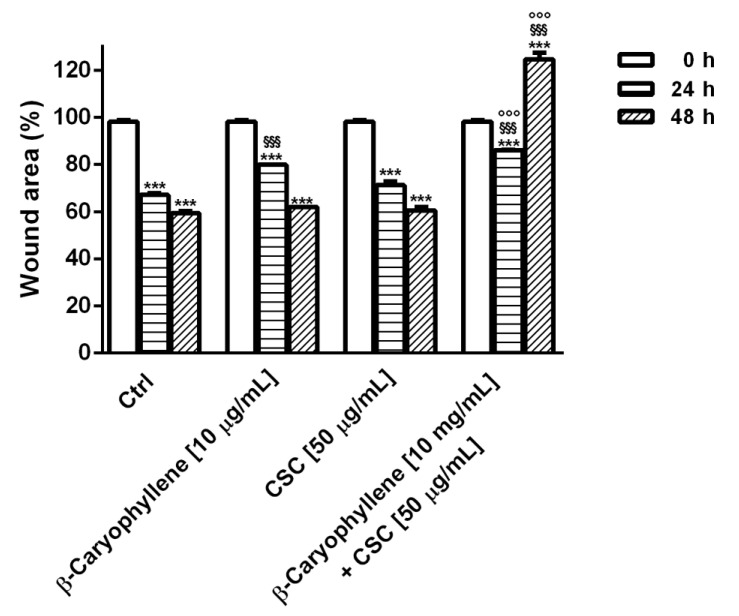
Quantification of MDA-MB-468 cells migration rate at 0 h, 4 h and 72 h (*n* = 3) after treatment with β-caryophyllene and cigarette smoke condensate (CSC). Data are displayed as mean ± SE of at least three independent experiments with at least three technical replicates (*n* = 9). *** *p* < 0.001, significant difference of each time point with respect to zero time (ANOVA + Dunnett’s multiple comparison post-hoc test). ^§§§^ *p* < 0.001, significant difference with respect to the control at the same time point (Student’s *t*-test). °°° *p* < 0.001, significant difference respect to CSC at the same time point (Student’s *t*-test).

In order to better outline the chemopreventive abilities of β-caryophyllene towards CSC, we also assessed the cell migration in the wound healing assay (Figure 14, Figure 15 and Figure S4). Images were captured at each time point and processed by Gen5™ Microplate Reader and Imager Software (Version 3.11, BioTek, AHSI, Milan, Italy) in order to highlight the wound area to be measured. Under our experimental conditions, the treatment of MDA-MB-468 cells with β-caryophyllene only slightly affected the cell migration, being the wound area similar to that of the control at each time point. Conversely, CSC lowered the wound area with respect to the control, especially after 48 h exposure, thus suggesting a promotion of migration abilities. Interestingly, this effect was blocked by the co-treatment with β-caryophyllene, which induced an increase in the wound area, especially after 48 h exposure.

**Figure 16:**
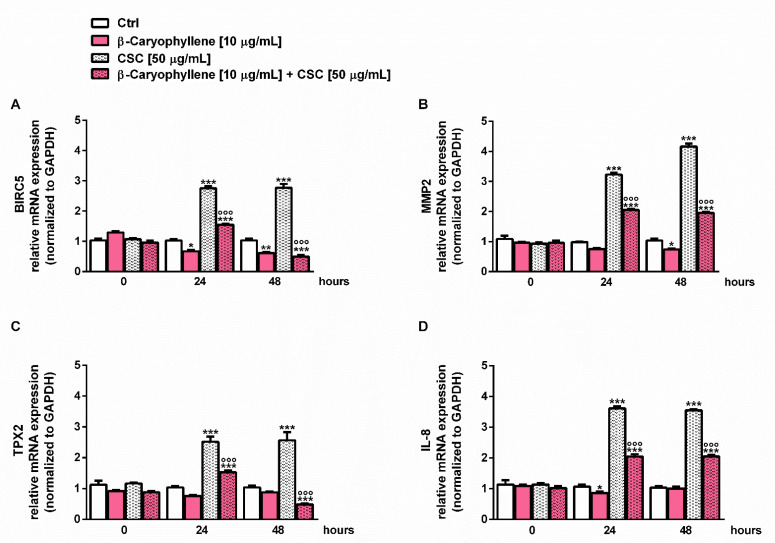
RTPCR analysis of cell migration factors BIRC5 (**A**), MMP2 (**B**), TPX2 (**C**), and IL-8 (**D**) induced by cigarette smoke condensate (CSC) and β-caryophyllene in MDA-MB-468 cancer cells after 24 h exposure. Data are displayed as mean ± SE of at least three independent experiments with at least three technical replicates (*n* = 9). * *p* < 0.05, ** *p* < 0.01 and *** *p* < 0.001, significant lowering of cell viability with respect to the control (ANOVA + Dunnett’s multiple comparison post-hoc test). °°° *p* < 0.001, significant difference respect to CSC (Student’s *t*-test).

Under the selected experimental conditions, we also evaluated the expression modulation of the genes involved in cell migration and metastasization, including BIRC5, MMP2, TPX2 and IL-8 at each time point (i.e., zero point and after 24 h and 48 h exposure). According to the results of the RT-qPCR analysis, we found that the expression of all of the assessed genes was markedly upregulated during incubation with CSC (Figure 16).

In particular, the expression of the BIRC5 gene was increased by CSC by almost three-fold after both 24 h and 48 h, while MMP2 was enhanced from at least three-fold to four-fold during incubation; similarly, the TPX2 and IL-8 genes were upregulated by 2.5-fold to 3.5-fold, respectively (Figure 16). Conversely, the presence of β-caryophyllene hindered the effects of CSC; indeed, the induced expression of the BIRC5 gene was halved after 24 h exposure, achieving a five-fold reduction after 48 h (Figure 16A). It is noteworthy that in this last condition, the gene expression of BIRC5 was also halved with respect to the basal level; accordingly, β-caryophyllene alone affected the basal level of the BIRC5 gene achieving a reduction by at least 1.5-fold after 24 h and 48 h exposure.

β-Caryophyllene also reduced the gene expression of MMP2 induced by CSC, achieving a lowering from 1.5- to 2-fold; moreover, it also induced a slight but significant reduction in the basal MMP2 expression after 48 h exposure (Figure 16B). Furthermore, the sesquiterpene lowered by 1.5- to 5-fold the gene expression of TPX2 induced by CSC (Figure 16C), and by almost two-fold that of IL-8 (Figure 16D), without affecting their basal levels. These findings support the results obtained in the migration assay and suggest that the inhibition of the MDA-MB-468 cancer cells proliferation and metastasization induced by CSC can arise from a gene regulation, as demonstrated by the modulation of the assayed genes and suggest an interest in clarifying the true mechanisms responsible for this regulation.

### 3.7. Modulation of STAT3 Activation

**Figure 17:**
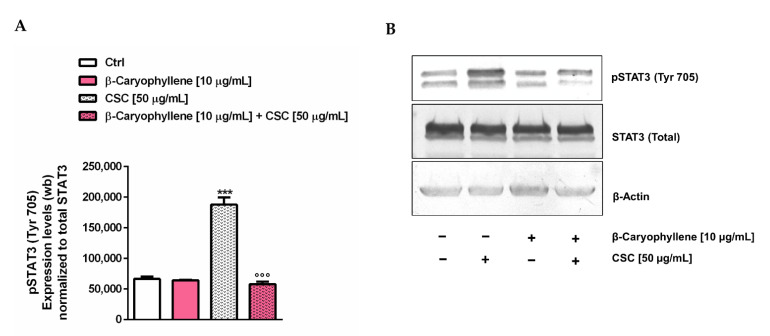
Effect of β-caryophyllene and cigarette smoke condensate (CSC) on the expression levels of phosphorylated STAT3 at tyrosine 705 residue in MDA-MB-468 cancer cells after 24 h exposure. (**A**) Densitometric bar graph analysis (data are expressed as mean ± standard error obtained from at least two independent experiments) (**B**) Representative Western blotting membrane, displaying phospho(Tyr705) STAT3, total STAT3 and β -actin, used as protein-loading controls. *** *p* < 0.001, significant lowering of cell viability with respect to the control (ANOVA + Dunnett’s multiple comparison post-hoc test). °°° *p* < 0.001, significant difference with respect to CSC (Student’s *t*-test).

Considering the well-known key role of the STAT3 cascade in the control of cell migration, cancer cell survival and apoptosis inhibition, especially through the activation by phosphorylation at tyrosine 705, namely pSTAT3(Tyr705) [37,48], we studied the modulation of this cascade as a possible chemopreventive mechanism of β-caryophyllene towards CSC. The results of the Western blotting analysis highlighted that pSTAT3(Tyr705) was markedly upregulated by the CSC treatment, with almost a three-fold increase with respect to the control (Figure 17, Figure S5 and Figure S6).

Conversely, β-caryophyllene completely suppressed the activation of pSTAT3(Tyr705), restoring its basal levels. These results suggest that the modulation by β-caryophyllene of the pSTAT3(Tyr705) activation induced by CSC can be responsible for the modulation of cell migration and apoptosis and probably for the inhibition of the autophagic fate induced by the natural sesquiterpene. Therefore, it could represent a key mechanism accounting for its chemopreventive power.

## 4. Discussion

In the present study, the chemopreventive properties of the natural sesquiterpene β-caryophyllene towards the damage induced by cigarette smoke condensate (CSC) in triple-negative breast cancer MDA-MB-468 cells were evaluated. The obtained results highlighted the ability of the sesquiterpene to interfere with events exploited by CSC to promote cell survival and chemoresistance, including genomic instability, cell cycle progress, autophagy, apoptosis, cell migration and related pathways. In particular, β-caryophyllene enhanced the rate of cell death, probably promoting oxidative stress, cell cycle arrest and apoptosis, and hindered cell recovery, autophagy activation and cell migration, which were probably activated by CSC as chemoresistant mechanisms. Interestingly, a marked inhibition of the CSC-induced STAT3 activation seems to represent a key mechanism of the chemoprevention by β-caryophyllene. The evidence that β-caryophyllene is endowed with chemopreventive properties agrees with previous results obtained in other in vitro models against smoke, cigarette butt extracts and other environmental pollutants [26,46,49,50]. Moreover, it has been highlighted that the natural sesquiterpene protected the noncancerous cholangiocytes towards the genotoxic damage induced by doxorubicin, despite enhancing the anticancer drug toxicity in the cholangiocarcinoma cells, thus strengthening the interest in this compound as a dual-acting chemopreventive and chemosensitizing agent [32].

Although cigarette smoke has been reported to contain several carcinogens, among which are the polycyclic aromatic hydrocarbons (e.g., benzo[a]pyrene) and N-nitrosamines [51,52], it is not fully understood which of them, and how, can be responsible for breast cancer exacerbation and chemoresistance development. At PME-GC/MS analysis, our CSC sample contained neophytadiene and nicotine as the most abundant identified components, along with minor components, including 7-methyl-6-tridecene, α-myrcene, triacetin, (Z)-2,6,10-trimethyl-1,5,9-undecatriene, 6,11-dimethyl-2,6,10-dodecatrien-1-ol and urs-12-en-28-ol. Neophytadiene is a tobacco diterpene which occurs naturally in tobacco and whose concentration increases upon curing and ageing [53]; it is regarded as a possible indicator of the smoke’s terpene content [54]. According to our results, it has been identified in other cigarette products (i.e., solid tobacco, cigarette smoke, cigarette papers and filters) [55,56]. However, there is a lack in the evidence available about its potential genotoxic power; conversely, anti-inflammatory properties have been highlighted in vitro [57]. Nicotine is a characteristic alkaloid of the tobacco plant and its derived products [58], which has been reported to induce genotoxicity and genome instability in different preclinical studies [59]; however, it also suppresses DNA synthesis and the proliferation of leukemic cells [60]. Although the true mechanisms involved remain to be clarified, genetic injury by nicotine seems to be partly due to oxidative stress, which in turn could be regulated by a nicotinic receptor-dependent pathway [61,62,63,64]. According to our results, high nicotine levels (55.8%) were found in CSC despite a lower relative amount (8.98%) in the CSE (cigarette smoke extract) sample. Among the minor components of our CSC samples, α-myrcene is a monoterpene commonly occurring in nature, especially in hops and cannabis [65], and formed during burning of the tobacco [66]; it is endowed with antioxidant and anti-inflammatory properties, and some studies reported lacking genotoxic effects and genoprotection against damage by tert-butyl hydroperoxide [67,68,69]. In one study, the genotoxic effects of myrcene in HepG2/C3A cells after metabolic activation were reported too [70]. Moreover, urs-12-en-28-ol is a pentacyclic triterpene, occurring in different plants and found to possess anti-inflammatory properties [71]. The other identified compounds (i.e., triacetin, 7-methyl-6-tridecene, (Z)-2,6,10-trimethyl-1,5,9-undecatriene and 6,11-dimethyl-2,6,10-dodecatrien-1-ol) are commonly reported as smoke additives or as metabolites produced during tobacco pyrolysis [62]. On the basis of the available evidence and considering the identified compounds, nicotine seems to potentially contribute to the genotoxicity and oxidative stress by CSC, although the involvement of other unidentified toxicants cannot be excluded. It is important to underline that CSC also contains some other compounds, such as myrcene and urs-12-en-28-ol, endowed with genoprotective and anti-inflammatory properties, which, however, fail to counteract the CSC damage in MDA-MB-468 cells.

In regard to the specific mechanisms accounting for the CSC injury in triple-negative breast cancer MDA-MB-468 cells, we found that cell death mainly resulted from mitochondrial damage, as highlighted by the MTT assay, and from cell membrane disruption, which led to LDH enzyme leakage: this last effect usually correlates with necrotic cell death [47]. As expected, the cytotoxic effects of CSC were associated with an increased oxidative stress, glutathione depletion and enhanced genome instability, as shown by the γH2AX levels. In particular, genotoxicity can be due to the increased oxidative stress, although other direct genotoxic mechanisms, ascribable to the toxicants contained in the smoke mixture, cannot be excluded. According to our results, DNA damage by cigarette smoke has been reported in different in vitro models, such as human cervical cancer cells and embryonic stem cells [72,73,74]; Albino et al. [72] showed that smoke condensate induced γH2AX in A549 human pulmonary adenocarcinoma cells and in normal human bronchial epithelial (NHBE) cells.

Along with the genotoxic effects, our results highlighted that CSC markedly increased the cell accumulation in the G1 phase, despite lowering the S and G2/M phases with respect to the control. In line with our hypothesis, Chai et al. [75] reported that cigarette smoke extract (CSE) increased the number of cells in the G1 phase and lowered the G2 one in a rat alveolar epithelial cell line; moreover, the G2 phase was also reduced by cigarette smoke in oral cancer cells [76]. These findings suggest an improved proliferative profile of CSC-treated MDA-MB-468 cells which can be reflected in the enhanced recovery abilities. In addition, the lowering of the S and G2/M phases suggests that CSC can impair the cell cycle checkpoints, thus allowing the cells to escape any control and proliferate in the presence of unrepaired DNA damages. Therefore, the induction of DNA damage by CSC along with the alteration in the cell cycle phases, probably consequent upon defective checkpoints, can be considered as events triggering of hyperproliferation and invasion of the MDA-MB-468 cells, and then chemoresistance.

When CSC was assessed in combination with β-caryophyllene, cytotoxicity rose, probably as a consequence of the genotoxic damage and oxidative stress; however, this effect seems to be mainly ascribable to a mitochondrial toxicity rather than necrosis, since the LDH release was not altered with respect to CSC. Interestingly, β-caryophyllene did not hinder the DNA damage by CSC, probably due to the failure of the DNA repair systems in the MDA-MB-468 cells, but markedly increased the G2/M cell cycle phase with respect to CSC. This evidence suggests that the cell cycle arrest induced by the sesquiterpene in the MDA-MB-468 cells can activate the checkpoints, which recognize the unsolved DNA damage induced by CSC and activate cell death, as was also found in other cancer cell models [77].

The increased cytotoxicity induced by β-caryophyllene in the CSC-damaged MDA-MB-468 cells can be also a consequence of the enhanced oxidative stress induced by the sesquiterpene. Oxidative stress plays an important role in cancer development and progression; indeed, despite normal cells, which usually grow under homeostatic conditions, characterized by low intracellular ROS levels, cancer cells are able to survive under altered redox balance and tolerate increased ROS levels, which also activate oncogenic signaling pathways and promote cancer cell proliferation, metastasis and chemoresistance [77]. This altered redox balance is impaired in the presence of excessive ROS levels, which overwhelmed the antioxidant systems and activated cell death [77]. Therefore, tolerable high ROS levels act as tumor-promoting factors, stimulating antioxidant cell defenses, while too high levels are tumor suppressive, since they disrupt the redox equilibrium and activate cell death. In line with this evidence, adding β-caryophyllene to CSC seems to trigger a tumor-suppressive oxidative stress, which is reflected in increased mitochondrial toxicity and apoptosis.

The HPLC analysis also highlighted that oxidative stress by CSC was reflected in glutathione depletion. It is noteworthy that, despite a marked lowering of the reduced GSH form, the oxidized GSSG one (namely glutathione disulfide) was only slightly affected by the treatment, thus suggesting that glutathione is depleted not only for the neutralization of ROS induced by CSC, but also for other purposes. Indeed, it is known that GSH is usually exploited by cells to scavenge electrophilic and oxidant species both directly, through quenching reactive hydroxyl free radicals, and indirectly, as a co-substrate of glutathione peroxidase: this leads to the formation of the oxidized GSSG form, which in turn can be reduced to two GSH molecules [78]. Moreover, it takes part in metabolizing processes, as a substrate for conjugation metabolic reactions of electrophilic endogenous compounds and xenobiotics, mediated by glutathione-S-transferases [78]. Therefore, we hypothesize that GSH depletion induced by CSC in the MDA-MB-468 cells can be due mainly to the smoke–carcinogen conjugation, which could be activated by cells as a primary defense line to counteract injury by toxicants; however, the true compound undergoing GSH conjugation remains to be clarified. For instance, α,β-unsaturated aldehydes (e.g., acrolein and crotonaldehyde) and α,β-unsaturated ketone (e.g., methyl vinyl ketone), which are known smoke carcinogens, are reported to form Michael-type adducts with glutathione, thus causing intracellular GSH depletion and cell deaths [79].

Usually, GSH depletion leads to different cell death types, including necrosis and necroptosis, apoptosis and autophagy, while its upregulation has been associated with an increased chemoresistance [80]: on the basis of this evidence, the altered GSH/GSSG ratio induced by CSC in the MDA-MB-468 cells seems to represent a buffering mechanism to avoid the acute toxicity of CSC, instead of sustaining cancer resistance. Despite the CSC effects, β-caryophyllene alone did not affect GSH/GSSG, while it restored the basal levels lowered by CSC. In this condition, increased antioxidant capacities of MDA-MB-468 cells should be expected; by contrast, the combination of β-caryophyllene and CSC increases cell death, suggesting that the increased GSH/GSSG can also be linked to other cell processes.

It is known that low GSH levels activate autophagy as an adaptive stress response [80], which has been reported to be a key feature of MDA-MB-468 cells [81]. In this respect, we found that CSC raised both autophagy levels, as revealed by the levels of autophagosomes, and apoptotic cell death with respect to the untreated cells. The increased autophagy triggering was also confirmed by the upregulation of the autophagic factors Beclin 1 and LC3 II. In the presence of β-caryophyllene, CSC-induced autophagy was partly lowered, while apoptotic cell death was retained; accordingly, the expression of Beclin 1 and LC3 II was reduced by the sesquiterpene with respect to CSC.

Autophagy is a catabolic process, activated by cells for recycling cytoplasmic constituents in autolysosomes under stress conditions, such as nutrient deficiency, oxidative stress, hypoxia and drug treatment; it is usually considered as a cell-protective mechanism; however, several pieces of evidence highlighted that excessive autophagy can also lead to cell death, thus suggesting that it represents a programmed and regulated process of self-elimination [82]. The process starts when the damaged intracellular components are encompassed by a double-membrane vesicle that leads to the autophagosome formation, which in turn is fused with lysosome to form an autolysosome; this last is responsible for intracellular material degradation and recycling products, which will be exploited by cells for metabolic purposes [83]. The entirety of the process includes five phases (i.e., initiation, elongation, maturation, fusion and degradation) which are regulated by key autophagy-related factors [83]. Among them, Beclin 1 is essential in the initiation phase, since it leads to the nucleation of autophagic vesicles, while LC3 (microtubule-associated protein 1 light chain 3) is involved in autophagosome elongation and maturation [83]. The last factor is available in a cytosolic LC3-I form which is converted in a LC3-phosphatidylethanolamine conjugate (LC3-II) recruited during autophagosome formation [83].

In cancer cells, autophagy has been reported to play a dual role, initially acting as a tumor suppressor process, and as promoting tumor progression during the advanced stages; an intricate link between autophagy and oxidative stress has also been reported [84]. Moreover, autophagy is strictly interconnected with apoptosis and different cross-talk between them have been highlighted depending on the type of cells [85]. Indeed, they can act as partners, leading to cooperative or synergistic effects, or with opposite trends, when autophagy blocks apoptotic cell death, thus promoting cell survival; at last, apoptosis may induce cell death both directly and through autophagy suppression [85].

Our results highlight that both autophagic and apoptotic cell death can contribute to the cytotoxic effects of CSC in the MDA-MB-468 cells. This could suggest a cooperating role of these processes to induce cell death, however, the results obtained with the co-treatment of β-caryophyllene and CSC highlighted that cytotoxicity increased when autophagy was partly lowered by the sesquiterpene, and the apoptotic rate was retained. In this context, our hypothesis is that apoptosis and autophagy are activated as opposite processes, leading apoptosis to cell death and autophagy to a buffering survival response. In support, autophagy-regulating mechanisms other than apoptosis, such as GSH depletion and genome instability, could be involved.

In the presence of β-caryophyllene, the autophagic rise induced by CSC in the MDA-MB-468 cells was partly hindered although apoptosis was retained, leading to the activation of other undefined cell death processes. According to our results, an upregulation of the autophagic cascade was postulated to be a mechanism by which CSC induces mitochondrial dysfunction in granulosa cells [86]. Similarly, a cigarette smoke extract induced autophagy and senescence in immortalized fibroblasts, which in turn promoted tumor growth when co-injected with cancer cells in animal models [87]. The autophagy-modulating effects of β-caryophyllene in cancer cells have been scantily investigated; a possible switch of autophagy to apoptosis has been hypothesized by Irrera et al. [88], since the sesquiterpene decreased the expression of Beclin-1, LC3 and p62/SQSTM1 in glioblastoma cells. Future more-in-dept studies could clarify the true mechanisms involved in the β-caryophyllene regulation of autophagy in triple-negative breast cancer cells and in other cancer models.

Under our experimental conditions, we also highlighted that CSC promoted the metastasizing abilities of the treated MDA-MB-468 cells. Indeed, although the cell migration was only slightly increased with respect to the control, the RT-PCR analysis showed an enhanced expression of the migration factors BIRC-5, MMP-2, TPX-2 and IL-8. This suggests that the acute exposure to CSC is able to promote the activation of cell migration, which could be reflected in the raised recovery abilities highlighted during the 72 h post treatment. According to our results, a recent study by Yu et al. showed that the substance markedly reduced the migration of human bladder cancer cells, and the effect was associated with a deregulation of the STAT-3/mTOR/AKT signaling. Moreover, we recently showed that β-caryophyllene oxide, a major metabolite of β-caryophyllene widely occurring in nature [26], also blocked migration of human pancreatic adenocarcinoma Bx-PC3 cells [41]. However, to the best of our knowledge, previous evidence about the ability of the sesquiterpene to hinder cancer cell migration induced by toxicants, such as cigarette smoke, is lacking.

Among the migration factors, BIRC-5 (also known as survivin) is known to regulate cell division and inhibit apoptosis through blocking caspase activation [89]. An aberrant upregulation of this factor has been found in different cancers, including breast cancers, in which it is associated with chemoresistance and poor prognosis [89]. Similarly, MMP-2 (also referred to as gelatinase A) has been found to be responsible for the invasion and metastasis of malignant melanoma and other cancers, such as breast cancers [90,91]. TPX2 (or targeting protein for Xklp2) is a microtubule protein involved in cell cycle control; indeed, it has been reported to regulate γ-H2AX signals during interphase. An overexpression of TPX2 has been highlighted in several malignancies, including breast cancer, pancreatic cancer and hepatocellular carcinoma [92,93]. At last, IL-8 is a chemotaxis cytokine, which has been reported to be upregulated in poor prognosis triple-negative breast cancers; it also promoted migration of the MDA-MB-231 cells via PI3K-Akt and MAPK cascades and induced cell cycle arrest in the S phase [94,95]. Our results agree with the literature about the basal expression of these factors in the MDA-MB-468 cells and highlighted their modulation as a mechanism accounting for the metastasizing effects of CSC. Noteworthy, we also found an S phase accumulation of untreated MDA-MB-468 cells, which can be explained by the IL-8 upregulation, as previously found [94].

The expression of these genes is correlated with the activation of the STAT3 (signal transducers and activators of transcription 3) cascade [89,90,96,97], which is known to promote cancer cell survival and cell migration, and to inhibit apoptosis especially after activation by phosphorylation at tyrosine 705 [48]. pSTAT3(Tyr705) has been found to be constitutively activated in different breast cancers, especially in triple-negative ones [98]. Its downregulation has also been associated with a reduced growth of different breast cancer cell subtypes [99,100], thus suggesting an interest in the STAT3 inhibitor as a potential anticancer or adjuvant strategy for breast cancer. Our results highlighted that CSC markedly activated pSTAT3(Tyr705) in MDA-MB-468 cells, but its effect was completely reversed by β-caryophyllene. This evidence suggests that blocking the CSC-induced pSTAT3(Tyr705) activation by the sesquiterpene can be reflected in the reduced expression of migration factors, leading to impaired migrating abilities; moreover, the apoptosis/autophagy crosstalk could be affected. Therefore, the interference with the STAT3 cascade by β-caryophyllene could represent a key mechanism accounting for its chemopreventive power. Previous evidence highlighted its ability to block the activation of pSTAT3(Tyr705) induced both by toxicants and drugs [26,32,41]; however, the true mechanism responsible for this effect is not already understood.

Altogether, the obtained results confirm the carcinogenic risk associated with cigarette smoke exposure and allow an explanation of the molecular mechanisms responsible for CSC-induced chemoresistance. Moreover, they strengthen the interest in β-caryophyllene and its analogues as chemopreventive strategies against environmental pollutants in diverse types of cancers.

## 5. Strengths, Limits and Future Perspectives

Breast cancer is one of the most common diseases in women, characterized by a marked phenotypic diversity and recurrence, which may lead to different and complicated chemoresistance mechanisms, many of which not fully elucidated, and in a diverse susceptibility to chemotherapy, chemoresistance and poor clinical outcomes [101,102,103,104]. Particularly, basal-like carcinomas, also known as triple-negative breast cancers (TNBC), are highly aggressive and metastasizing subtypes, with limited effective treatment strategies [105,106]. This scenario can be exacerbated by exposure to environmental pollution, which was reported to promote the metastasizing power and chemoresistance of different cancer cells [9,107,108,109,110]. In particular, smoking habits have been associated with an increased risk of mortality in breast cancer patients, probably due to a lower chemotherapy susceptibility and chemoresistance development [111,112,113]. In this context, along with early diagnosis, a great interest has been devoted towards chemopreventive agents, which can be exploited by both healthy and oncological subjects to prevent or repair CS damage, thus limiting the occurrence and aggressiveness of breast cancers. Preclinical studies highlighted the possible benefits of anti-inflammatory drugs, dietary polyphenols and other natural compounds against the oxidative and inflammatory damage by cigarette smoke, however limited evidence about their ability to hinder cancer progression and chemoresistance development is currently available [26,114,115,116,117,118,119], and the true mechanisms involved remain to be elucidated. In this context, our results provide additional evidence about the mechanisms implicated in the chemoresistance induced by cigarette smoke in triple-negative breast cancer cells, particularly the increased proliferative abilities, probably due to a lowered control in G2/M phase, the promotion of autophagic cell death and migration, along with the STAT3 activation, which seem to be activated as buffering processes to promote the cancer cell resilience to the acute CSC damage, thus resulting in the increased recovery abilities.

β-Caryophyllene resulted in being able to block most of these events, leading to the activation of programmed cell death. It is noteworthy that this effect seems to result from different processes, including the activation of cell cycle checkpoints, which block the proliferation of CSC-damaged cells, the increased oxidative stress, which achieves tumor-suppressing levels, the modulation of the autophagic cell death, probably associated with the recovery of the basal GSH levels, and of the migration factors, which underpin the metastasizing abilities of the MDA-MB-468 cells. Another interesting point to be underlined is the evidence of a peculiar crosstalk between autophagy and apoptosis in the MDA-MB-468 cells, which seem activated as opposite processes, leading apoptosis to cell death and autophagy to a buffering survival response. This represents an important finding to also be considered during chemotherapeutic regimens, to limit the acquired resistance induced by anticancer drugs, which often impair their therapeutic efficacy.

Despite the advanced knowledge about the chemoresistance in MDA-MB-468 cells and the possible chemoprevention by β-caryophyllene, several issues, including the key carcinogenic compounds of CSC, the unknown cell death processes activated by the sesquiterpene along with those induced by CSC, the true mechanisms by which it regulates autophagy along with the STAT3 cascade, and the involvement of other crucial processes triggering chemoresistance and cancer aggressiveness, remain to be clarified. Moreover, confirming the obtained results in other cancer cell lines, particularly in different breast cancer subtypes, and in vivo could strengthen the interest in the chemopreventive properties of β-caryophyllene towards cigarette smoke, thus underpinning its clinical application. Lastly, considering the limited solubility of sesquiterpene in biological fluids, developing suitable delivery systems can improve its biological responses, also favoring its pharmaceutical development.

## 6. Conclusions

Growing evidence highlighted an interest in the natural sesquiterpene β-caryophyllene as a chemopreventive agent due to its multiple and pleiotropic bioactivities. Although further in vivo studies are required in confirmation, our results strengthen this interest and highlight that the substance was able to counteract the harmful effects and chemoresistance induced by cigarette smoke in triple-negative breast cancer cells, thus suggesting a possible interest in the substance as a preventive or adjuvant strategy, to be exploited not only in healthy subjects but also in cancer patients. Moreover, they contribute to a better understanding of the mechanisms accounting for cigarette smoke cancer chemoresistance and metastasis and suggest the need to avoid the exposure to smoke in breast cancer patients, due to its negative impact on their survival chances. Altogether, the present findings encourage further studies to clarify some unanswered questions, including the in vivo efficacy and bioavailability issues, in order to support the pharmaceutical development of β-caryophyllene as a chemopreventive agent.

## Figures and Tables

**Table 1:** List of oligonucleotides, general conditions, and validation parameters used to perform RT-qPCR.

Gene	Brand	Primer (5′ → 3′)		Annealing (°C)	Efficiency (%)	R^2^
BIRC-5	Bio-Rad	Forward	N/A (Cod. qHsaCED0001615)	60	97.0	0.999
		Reverse				
MMP2	Bio-Rad	Forward	N/A (Cod. qHsaCED0042560)	60	99.0	0.999
		Reverse				
TPX2	Bio-Rad	Forward	N/A (Cod. qHsaCID0016024)	60	98.0	0.997
		Reverse				
IL-8	Bio-Rad	Forward	N/A (Cod. qHsaCED0023767)	60	97.0	0.999
		Reverse				
GAPDH	Bio-Rad	Forward	N/A (Cod. qHsaCED0038674)	60	97.0	0.999
		Reverse				

**Table 2:** Chemical components (relative *%*) of cigarette smoke condensate (CSC) identified at SPME-GC/MS analysis.

No.	Component ^1^	LRI ^2^	LRI ^3^	Smoke (%) ^4^
1	α-Myrcene	979	981	0.8
2	Triacetin	1340	1344	1.2
3	Nicotine	1366	1360	26.7
4	(Z)-2,6,10-Trimethyl-1,5,9-undecatriene	1410	*	2.4
5	7-Methyl-6-tridecene	1455	*	6.9
6	6,11-Dimethyl-2,6,10-dodecatrien-1-ol	1665	1658	3.1
7	Neophytadiene	1912	1915	56.4
8	Urs-12-en-28-ol	3385	3376	2.6
	SUM (%)			99.9

^1^ The components are reported according to their elution order on a polar column; ^2^ Linear Retention index (LRI) measured on a polar column; ^3^ LRI from literature; ^4^ Percentage mean values of CSC components; *: LRI not available.

**Table 3:** IC_50_ values of β-caryophyllene, cigarette smoke condensate (CSC) and the positive control doxorubicin in triple-negative breast cancer MDA-MB-468 cells. Data represent the mean ± SE of at least three independent experiments with at least three technical replicates (*n* = 9).

Tested Sample	IC_50_ [µg/mL] (CL)
	MDA-MB-468
CSC	134.8 (119.6–152.7) ***
β-Caryophyllene	18.6 (15.9–21.7) ***
Doxorubicin	4.7 (3.8–6.7)

CL, confidence limits. *** *p* < 0.001, significantly different than doxorubicin (Student’s *t*-test).

**Table 4:** Semiquantitative analysis of LC3B and Beclin-1 expression induced by β-caryophyllene and cigarette smoke condensate (CSC) in MDA-MB-468 cancer cells after 24 h exposure. Analysis has been carried out (five fields for each treatment) using a previous published grading system [42]: 0%–5% = negative; 6–10% = +/−; 11–30% = +; 31–60% = ++; >61% = +++.

	Ctrl	β-Caryophyllene	CSC	β-Caryophyllene + CSC
LC3B	-	+/−	+	+/−
Beclin-1	-	+/−	+	+/−

## Data Availability

Not applicable.

## Supplementary Materials

The following are available online at https://www.mdpi.com/article/10.3390/biomedicines10092257/s1. Figure S1. Effect of the co-treatment of β-caryophyllene and CSC in triple-negative breast cancer MDA-MB-468 cells after 24 h exposure and subsequent 72 h cell recovery; Figure S2. Modulation of phosphorylated H2AX histone (γH2AX) by CSC 50 µg/mL, β-caryophyllene 10 µg/mL and their combination in triple-negative breast MDA-MB-468 cells under pre, co- and post-treatment protocols; Figure S3. Apoptosis extent after a 3 h exposure to β-caryophyllene 10 µg/mL, CSC 50 µg/mL, and their combination in triple-negative breast MDA-MB-468 cells; Figure S4. Original high contrast brightfield images (4 × magnification) of wound healing in triple-negative breast MDA-MB-468 cells were treated with CSC 50 µg/mL, β-caryophyllene 10 µg/mL and their combination; Figure S5. Original Western blotting membrane used to evaluate the expression levels of STAT3 phosphorylation at tyrosine 705 residue in triple-negative breast MDA-MB-468 cells. Figure S6. Original Western blotting membrane used to evaluate the expression levels of total STAT3 and β-actin in triple-negative breast MDA-MB-468 cells.
