# Supplementary material for: β-Caryophyllene Counteracts Chemoresistance Induced by Cigarette Smoke in Triple-Negative Breast Cancer MDA-MB-468 Cells
Source: Biomedicines. 2022 Sep 12;10(9):2257. doi: 10.3390/biomedicines10092257 (PMC9496176; doi:10.3390/biomedicines10092257)
Supplement: Supplementary file 1 [file biomedicines-10-02257-s001.zip › biomedicines-1866646-supplementary.pdf]

Supplementary Materials

β-Caryophyllene Counteracts Chemoresistance Induced by Cigarette Smoke in Triple-Negative Breast Cancer MDA-MB-468 Cells

Antonella Di Sotto [1,*], Marco Gullì [1], Marco Minacori [2], Romina Mancinelli [3], Stefania Garzoli [4], Ester Percaccio [1], Alessio Incocciati [2], Donatella Romaniello [2,5], Gabriela Mazzanti [1,*], Margherita Eufemi [2] and Silvia Di Giacomo [1]

[1] Department of Physiology and Pharmacology "V. Erspamer", Sapienza University of Rome, Piazzale Aldo Moro 5, 00185 Rome, Italy
[2] Department of Biochemical Science "A. Rossi Fanelli", Sapienza University of Rome, Piazzale Aldo Moro 5, 00185 Rome, Italy
[3] Department of Anatomical, Histological, Forensic and Orthopedic Sciences, Sapienza University of Rome, Piazzale Aldo Moro 5, 00185 Rome, Italy
[4] Department of Chemistry and Technology of Drugs, Sapienza University of Rome, Piazzale Aldo Moro 5, 00185 Rome, Italy
[5] Department of Experimental, Diagnostic and Specialty Medicine, University of Bologna, 40138 Bologna, Italy
* Correspondence: antonella.disotto@uniroma1.it (A.D.S.); gabriela.mazzanti@uniroma1.it (G.M.)

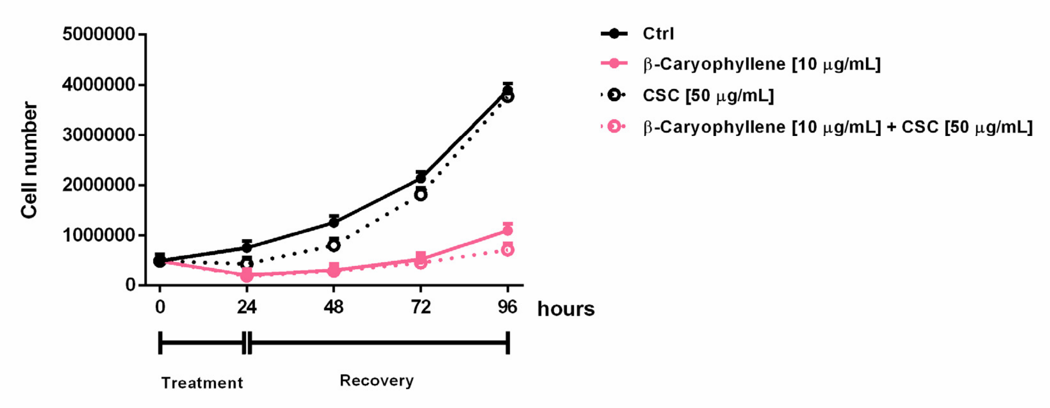

Figure S1. Effect of the co-treatment of β-caryophyllene and cigarette smoke condensate (CSC) in triple negative breast cancer MDA-MB-468 cells after 24 h exposure and subsequent 72 h cell recovery. Cell number was determined by trypan blue exclusion assay at each time point. Data are displayed as mean ± standard error (SE) of at least two experiments with at least three technical replicates (*n* = 6).

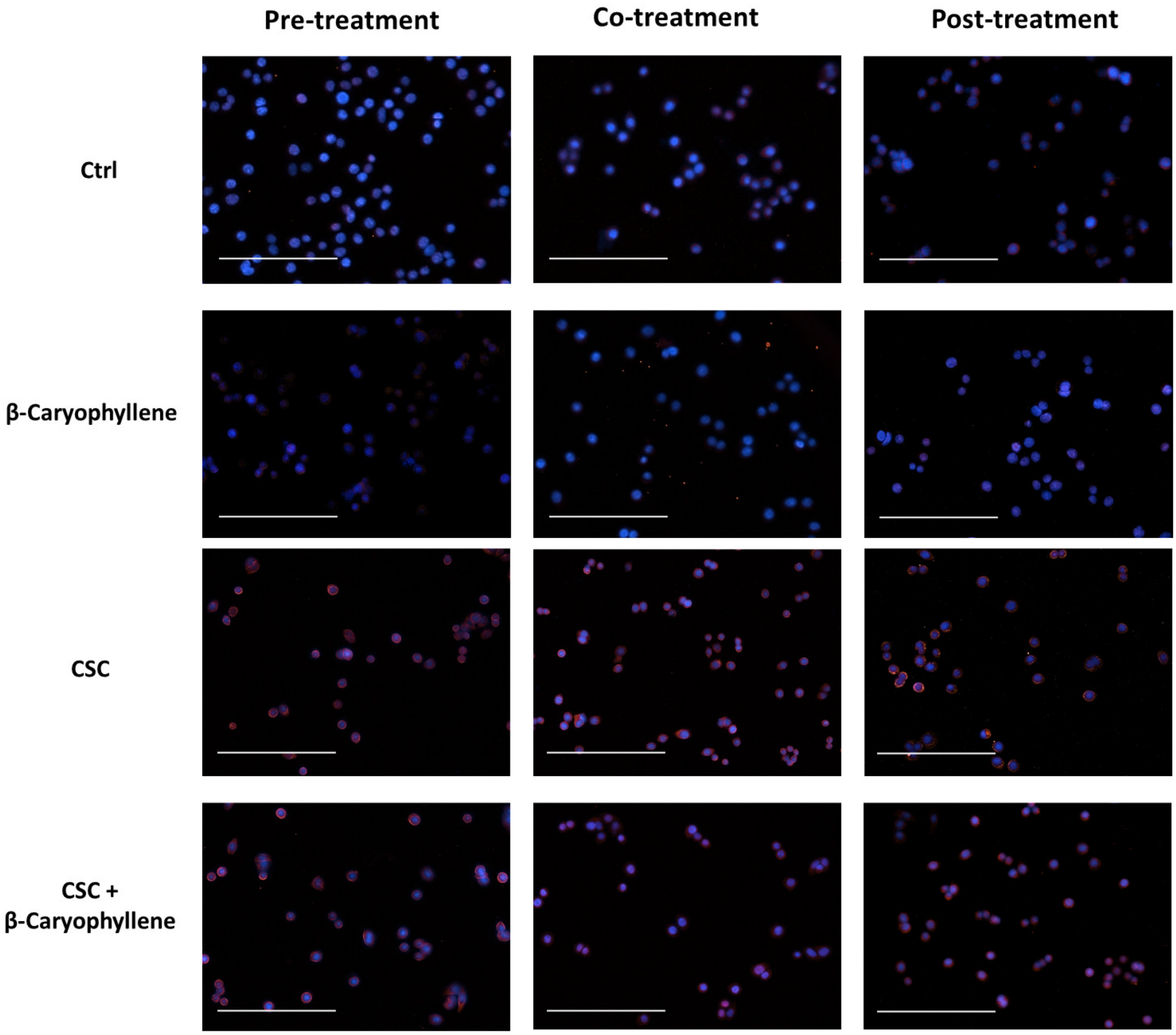

Figure S2. Modulation of phosphorylated H2AX histone (γH2AX) by cigarette smoke condensate (CSC) 50 μg/mL, β-caryophyllene 10 μg/mL and their combination in triple negative breast MDA-MB-468 cells under pre, co- and post-treatment protocols. Representative images of cells, stained by an Alexa Fluor® 647 anti-gamma H2A.X (phospho S139) antibody and Hoechst 33258, were obtained at immunofluorescence analysis (original 10X magnification) using a Cytation 1 Cell Imaging Multimode Reader (Biotech, USA) and processed by Gen5™ Microplate Reader and Imager Software 3.11. Scale bars = 200 μm.

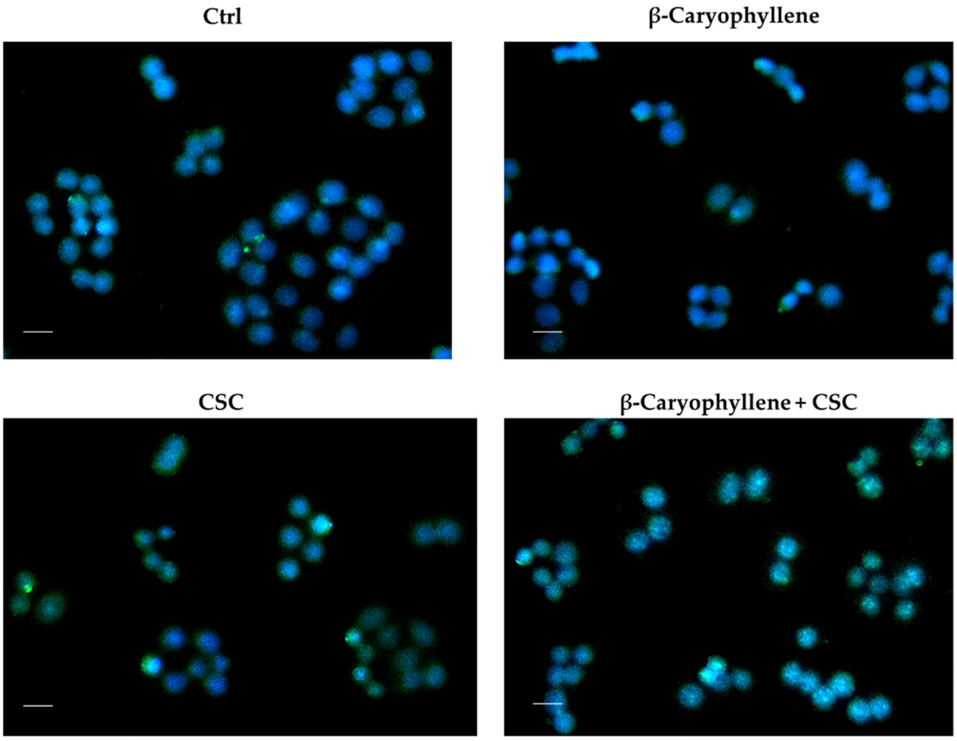

Figure S3. Apoptosis extent after a 3 h exposure to β-caryophyllene 10 μg/mL, cigarette smoke condensate (CSC) 50 μg/mL, and their com-bination in triple negative breast MDA-MB-468 cells. Representative images of cells, stained by Hoechst 33258/ Annexin-V-FITC, were obtained at immunofluorescence analysis (original 10X magnification) using a Cytation 1 Cell Imaging Multimode Reader (Biotech, USA) and processed by Gen5™ Microplate Reader and Imager Software 3.11. Scale bars = 20 μm.

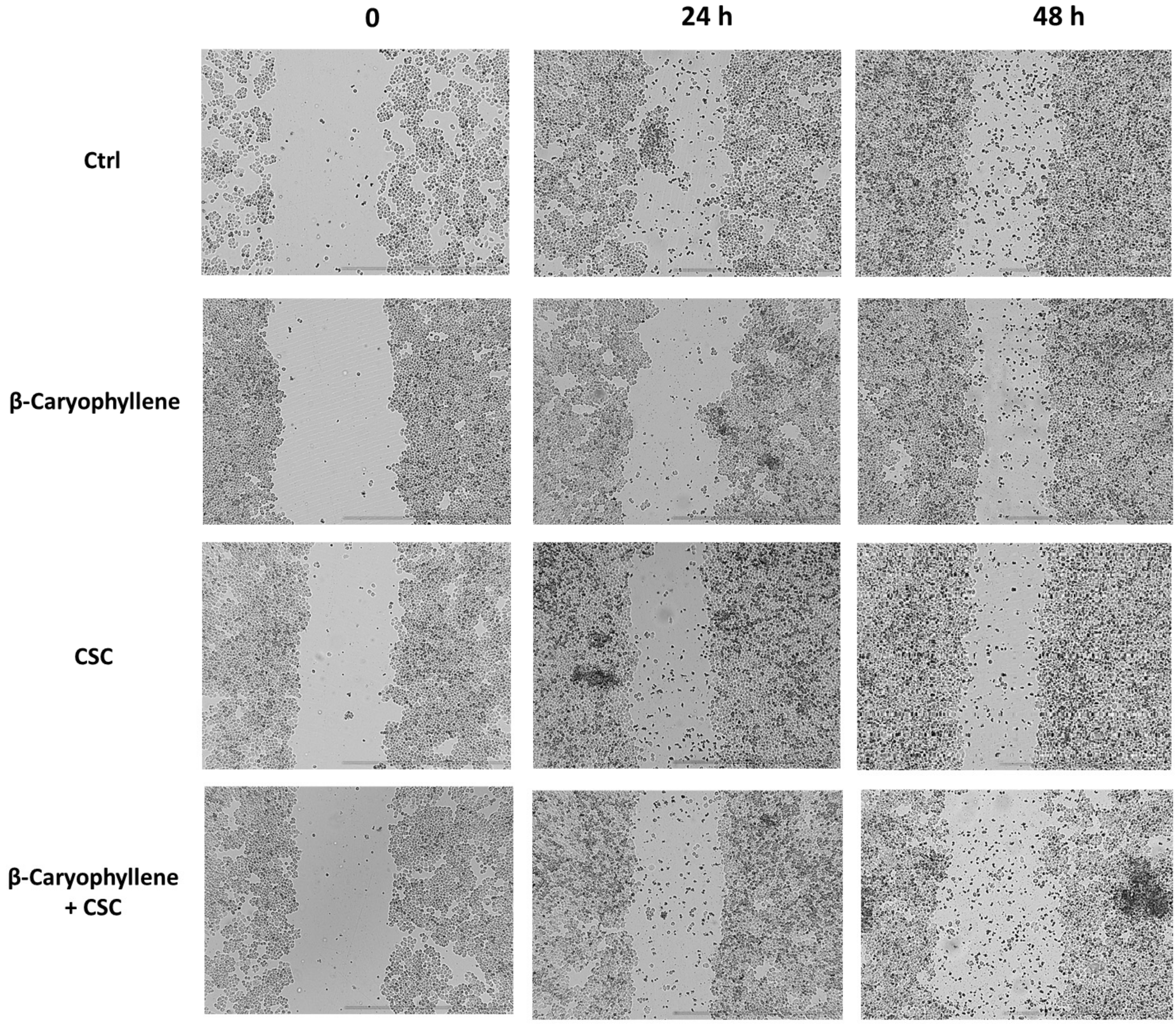

Figure S4. Original high contrast brightfield images (4X magnification) of wound healing in triple negative breast MDA-MB-468 cells were treated with cigarette smoke condensate (CSC) 50 μg/mL, β-caryophyllene 10 μg/mL and their combination. Images were captured at zero time and after 24h and 48 h exposure, using a Cytation 1 Cell Imaging Multimode Reader (Biotech, USA) and processed by Gen5™ Microplate Reader and Imager Software 3.11. Scale bars = 1000 μm.

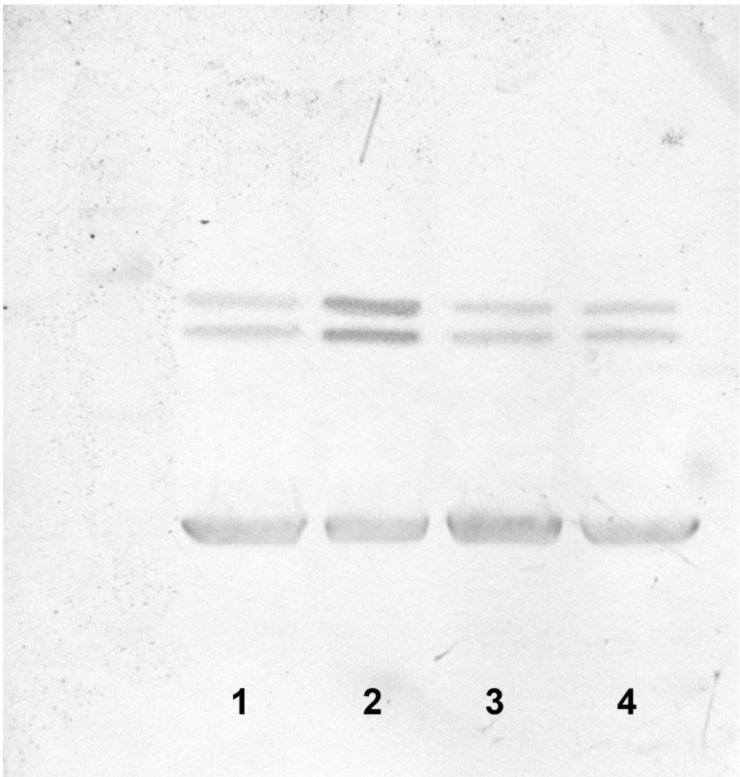

Figure S5. Original Western blotting membrane used to evaluate the expression levels of STAT3 phosphorylation at tyrosine 705 residue in triple negative breast MDA-MB-468 cells. 1) Control; 2) cigarette smoke condensate (CSC) 50 μg/mL; 3) β-caryophyllene 10 μg/mL; 4) β-caryophyllene 10 μg/mL + CSC 50 μg/mL.

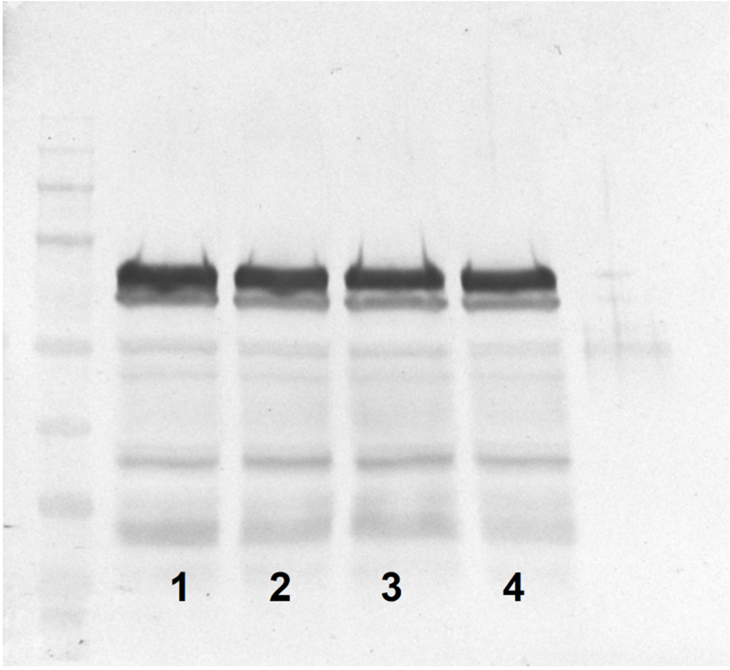

Figure S6. Original Western blotting membrane used to evaluate the expression levels of total STAT3 and β-actin in triple negative breast MDA-MB-468 cells. 1) Control; 2) cigarette smoke condensate (CSC) 50 μg/mL; 3) β-caryophyllene 10 μg/mL; 4) β-caryophyllene 10 μg/mL + CSC 50 μg/mL.
